# Comprehensive Investigation of Polymorphic Stability and Phase Transformation Kinetics in Tegoprazan

**DOI:** 10.3390/pharmaceutics17070928

**Published:** 2025-07-18

**Authors:** Joo Ho Lee, Ki Hyun Kim, Se Ah Ryu, Jason Kim, Kiwon Jung, Ki Sung Kang, Tokutaro Yamaguchi

**Affiliations:** 1 J2Hbiotech Inc., #210, B Dong, Suwon Venture Valley II, Suwon 16648, Republic of Korea; shadowjhl@empal.com (J.H.L.); fbtpdk0917@gmail.com (S.A.R.); jsbach@j2hbio.com (J.K.); pharmj@j2hbio.com (K.J.); 2 School of Pharmacy, Sungkyunkwan University, Suwon 16419, Republic of Korea; khkim83@skku.edu; 3 College of Pharmacy, Seoul National University, Seoul 08826, Republic of Korea; 4 College of Korean Medicine, Gachon University, Seongnam 13120, Republic of Korea

**Keywords:** Tegoprazan, polymorphism, disappearing polymorph, solvent-mediated phase transformation (SMPT), conformational analysis, KJMA equation, DFT-D, crystal structure prediction (CSP)

## Abstract

**Background/Objectives:** Tegoprazan (TPZ) is a potassium-competitive acid blocker (P-CAB) used to treat conditions such as gastroesophageal reflux disease, peptic ulcer, and *Helicobacter pylori* infection. It exists in three solid forms: amorphous, Polymorph A, and Polymorph B. This study investigates the molecular basis of polymorph selection, focusing on conformational bias and solvent-mediated phase transformations (SMPTs). **Methods:** The conformational energy landscapes of two TPZ tautomers were constructed using relaxed torsion scans with the OPLS4 force field and validated by nuclear Overhauser effect (NOE)-based nuclear magnetic resonance (NMR). Hydrogen-bonded dimers were analyzed using DFT-D. Powder X-ray diffraction (PXRD), differential scanning calorimetry (DSC), solubility, and slurry tests were conducted using methanol, acetone, and water. Kinetic profiles were modeled with the Kolmogorov–Johnson–Mehl–Avrami (KJMA) equation. **Results:** Polymorph A was thermodynamically stable across all analyses. Both amorphous TPZ and Polymorph B converted to A in a solvent-dependent manner. Methanol induced direct A formation, while acetone showed a B → A transition. Crystallization was guided by solution conformers and hydrogen bonding. **Conclusions:** TPZ polymorph selection is governed by solution-phase conformational preferences, tautomerism, and solvent-mediated hydrogen bonding. DFT-D and NMR analyses showed that protic solvents favor the direct crystallization of stable Polymorph A, while aprotic solvents promote the transient formation of metastable Polymorph B. Elevated temperatures and humidity accelerate polymorphic transitions. This crystal structure prediction (CSP)-independent strategy offers a practical framework for rational polymorph control and the mitigation of disappearing polymorph risks in tautomeric drugs.

## 1. Introduction

The term “disappearing polymorphs” describes situations in which a previously reported crystalline form becomes irreproducible over time, often coinciding with the emergence of a new polymorphic form [1,2,3,4,5]. This issue is particularly critical in the pharmaceutical industry, as it can significantly impact the stability, manufacturability, and regulatory compliance of drug products. Several well-known pharmaceutical compounds—including ritonavir, paroxetine hydrochloride hemihydrate, and loxoprofen sodium hydrate—have been subject to product recalls due to polymorphic transitions [6,7]. More recently, in 2023, spontaneous crystallization was observed in certain bottles of cyclosporine oral solution (Sandimmune^®^, Novartis, Basel, Switzerland), ultimately resulting in a product recall in 2024 due to concerns over content uniformity [8].

The primary cause of disappearing polymorphs is generally attributed to spontaneous transformation into a thermodynamically more stable form [9,10]. As crystalline solids tend to evolve toward more stable packing arrangements over time, the initially discovered polymorph may not represent the most stable form. Trace contamination with seed crystals or partial dissolution followed by recrystallization during storage can trigger such polymorphic conversions, rendering the original form irreproducible. Polymorphism profoundly affects the physicochemical properties of active pharmaceutical ingredients (APIs), including solubility, dissolution rate, chemical stability, and bioavailability. Therefore, identifying and controlling solid-state forms is essential for ensuring product quality, reproducibility, and regulatory robustness.

While solid-state screening during early-phase drug development is a well-established strategy, predicting the most stable polymorph under real-world conditions remains a formidable challenge. Computational tools such as CSP have shown significant progress in recent years [5,11,12,13,14,15], but their practical utility is still constrained by factors such as conformational flexibility, solvation effects, and tautomerism. In particular, for flexible drug-like molecules with multiple torsional degrees of freedom and tautomeric states, CSP becomes computationally expensive and error-prone. Solvent-mediated effects, which are typically not included in CSP protocols, can shift conformational populations and alter crystallization pathways, thus undermining the reliability of in silico predictions.

TPZ is a P-CAB approved in several Asian countries for the treatment of acid-related gastrointestinal disorders [16,17]. Unlike traditional proton pump inhibitors (PPIs), P-CABs offer a faster onset and longer duration of acid suppression. Three solid-state forms of TPZ have been reported: an amorphous form, Polymorph A, and Polymorph B [18,19,20]. Polymorph A is used in marketed formulations (e.g., K-CAB^®^ in Korea and Taiwan) due to its superior stability. However, the transient formation of metastable forms during processing or improper storage may affect product consistency.

The commercial formulation of TPZ employs Polymorph A, which has been identified as the most thermodynamically stable form under both ambient and accelerated storage conditions. Its robust physical and chemical stability makes it suitable for pharmaceutical use. However, during manufacturing or storage, the transient formation of the amorphous state or Polymorph B has occasionally been observed under certain solvent-mediated conditions. These metastable forms may convert to Polymorph A over time, raising concerns regarding reproducibility, batch-to-batch consistency, and solid-state characterization—particularly considering the compound’s high conformational and tautomeric flexibility in solution.

Our previous work confirmed the existence of Polymorphs A and B through structure determination based on PXRD patterns obtained from multiple crystalline samples of TPZ exhibiting distinct diffraction profiles. These samples were collected from independent sources and analyzed using structure determination by powder diffractometry (SDPD), allowing unambiguous assignment to Polymorph A and Polymorph B. While Polymorph A was found to be thermodynamically more stable, Polymorph B appeared only under specific crystallization conditions and disappeared upon prolonged exposure, suggesting characteristics of a disappearing polymorph.

The sensitivity of TPZ to its solution environment makes these transformations particularly relevant in manufacturing contexts. Although Polymorph A is the intended and preferred form, the transient presence of metastable phases during dissolution or crystallization necessitates rigorous control strategies to ensure product quality and regulatory compliance.

From a molecular perspective, both polymorphs exhibit crystal packing arrangements stabilized by intermolecular interactions, including hydrogen bonding, π–π stacking, and π–H interactions. Under accelerated conditions (40 °C/75% RH), both the amorphous form and Polymorph B were observed to convert into Polymorph A within approximately eight weeks, as confirmed by PXRD [20] (see also Section S1). These observations raise the question of whether such transformations represent typical polymorphic conversions, involve unknown intermediate forms, or reflect alternative crystallization pathways. To date, no additional crystalline forms of TPZ have been identified beyond the known three.

TPZ’s structural flexibility, tautomerism, and conformational freedom pose significant challenges for both experimental and theoretical studies. With 47 non-hydrogen atoms, multiple rotatable bonds, and at least two major tautomeric forms, TPZ represents a nontrivial case for polymorph prediction and control. In this context, we hypothesized that solution-phase conformational preferences and intermolecular interactions—particularly hydrogen bonding—play decisive roles in the selection of the final polymorphic form during crystallization.

The objectives of this study are twofold: (1) to examine whether solution-state conformational bias can explain the preferential crystallization of Polymorph A and (2) to assess whether simplified computational approaches can complement traditional polymorph screening methods. To achieve these goals, we constructed conformational energy landscapes using relaxed torsion scans based on the OPLS4 force field (as implemented in Schrödinger MacroModel), exploring two key dihedral angles in 10° increments for each tautomeric form. Boltzmann-weighted probabilities were calculated from the relative energies and compared with experimental solution structures derived from NOE-based NMR spectra. Two dominant conformers in solution were found to correspond closely to the packing motif of Polymorph A.

To evaluate the role of intermolecular forces in solid-state selection, hydrogen-bonded dimers derived from the crystal structures of Polymorphs A and B were extracted and subjected to single-point energy calculations using density functional theory with empirical dispersion corrections (wB97X-D3(BJ)/def2-TZVPP). These calculations indicated that the hydrogen-bonding network present in Polymorph A is energetically more favorable than that of Polymorph B, reinforcing the hypothesis that hydrogen bonding governs the polymorphic outcome.

Experimentally, we investigated the phase behavior of TPZ through solubility measurements, DSC, and time-dependent PXRD. The absence of a glass transition temperature and the presence of thermal events only above 100 °C suggested that phase transformation does not proceed via solid-state transitions but rather through a solvent-mediated mechanism. Slurry experiments monitored by PXRD confirmed the conversion of both the amorphous form and Polymorph B into Polymorph A over time. This process was categorized as an SMPT [21,22,23], and its kinetics were evaluated using the KJMA model to derive empirical rate parameters [24,25]. Additional solubility monitoring and PXRD analysis revealed no anomalous behavior or evidence of unknown polymorphs, confirming that only the three known forms participate in the observed transitions.

In summary, this study explores the thermodynamic and kinetic determinants of polymorph selection in TPZ, integrating theoretical and experimental techniques to elucidate the roles of conformational flexibility, solvation, and hydrogen bonding. Our findings provide a complementary framework to CSP for guiding polymorph control in flexible drug molecules while also offering mechanistic insights into disappearing polymorph phenomena.

## 2. Materials and Methods

### 2.1. Materials

TPZ Polymorph A was obtained from HK inno.N Corporation (Seoul, Republic of Korea), which is also the supplier of the API used in the commercial formulation. Polymorph B was procured as a crystalline bulk material from Anhui Haoyuan Pharmaceutical Co., Ltd. (Anhui, China), and its identity was confirmed by PXRD prior to use. The amorphous form of TPZ was provided by Lee Pharma Limited (Hyderabad, Telangana, India). Although the specific preparation method was not disclosed, the material was confirmed to be amorphous based on the absence of diffraction peaks in PXRD and the absence of melting transitions in DSC. All materials were used as received, without further in-house processing or recrystallization. Each solid form was verified for identity and phase purity using PXRD and DSC before subsequent experiments. Although amorphous TPZ was initially obtained from three different suppliers and all confirmed to be amorphous by PXRD, slight differences in transformation kinetics were observed during preliminary screening. Due to limited and inconsistent material availability, a single representative batch was selected for all detailed kinetic analyses. Therefore, batch-to-batch or supplier-dependent variations were not systematically investigated. The purity of the materials, as reported in the suppliers’ Certificates of Analysis, was >99% for all three solid forms. No further purification was performed prior to experimentation.

### 2.2. Computational Methods for Conformational and Energetic Analysis

Molecular and crystal structure visualizations, as well as input preparation and analysis of molecular packing, were performed using Mercury version 2024.3.1 (Build 428097), Olex2 version 1.5-alpha, VESTA version 3.90.1a, and Avogadro version 1.2.0.

All energy landscape analyses and density functional theory with dispersion correction (DFT-D) calculations were conducted using the Jaguar version 11.9, release 128 (Schrödinger Suite 2023-1). Relaxed coordinate scans were performed with the OPLS4 force field to explore conformational space. Geometry optimizations were carried out at the DFT-D level [wB97X-D3(BJ)/6-31G**] using the Jaguar module, and single-point energy evaluations were performed at the same level using the def2-TZVPP basis set. Solvent effects were included using the polarizable continuum model (PCM), with chloroform specified as the dielectric medium.

### 2.3. NMR Measurements

^1^H, ^19^F, and ^13^C NMR spectra of crystalline forms A and B were recorded on a JEOL JNM-ECX400II spectrometer (JEOL Ltd., Tokyo, Japan) (^1^H: 400 MHz, ^13^C: 100 MHz, ^19^F: 376 MHz) at room temperature (25 °C), with CDCl_3_ and DMSO-d_6_ used as solvents, and tetramethylsilane (TMS) served as the internal reference for ^1^H and ^13^C spectra. For ^19^F NMR, trifluoroacetic acid (CF_3_COOH) was used as an external reference and calibrated to −76.55 ppm. All NMR data were processed using MestReNova software (version 14.2.1-27684; Mestrelab Research, 2021).

### 2.4. Solubility Measurement

Supersaturated solutions of TPZ Polymorphs A and B and the amorphous form were prepared as 150 mL slurries using acetone, methanol, and water as solvents. Each slurry was transferred to the sample holder of a shaking incubator (LSI-150M, Gyeonggi, South Korea) and maintained at 20.0 ± 0.5 °C. The temperature was precisely controlled to minimize ambient fluctuations and ensure reproducibility, given that solubility is highly sensitive to even small temperature variations (e.g., 5 °C). Although 25 °C is commonly used, 20 °C was selected to reduce sample consumption during repeated sampling.

At each sampling point, approximately 300–500 μL of the solution was withdrawn using a micropipette and transferred into a 1 mL Eppendorf tube. The samples were immediately centrifuged at 15,000 rpm for 1–2 min and filtered through a 0.2 μm pore size membrane filter. The filtrate was then diluted as necessary and prepared for high-performance liquid chromatography (HPLC) analysis [26].

The quantification of dissolved TPZ was performed using an Agilent 1260 Infinity II HPLC system (Agilent Technologies, Santa Clara, CA, USA) equipped with Agilent Poroshell C18 column (150 mm × 4.6 mm, 2.7 μm) maintained at 30 °C. The mobile phase consisted of a 10 mM ammonium acetate buffer (pH 6.5) with 5% acetonitrile (Mobile Phase A) and pure acetonitrile (Mobile Phase B). Gradient elution was applied, beginning with 88% A and 12% B, ramping up to 90% B, and then returning to initial conditions. The flow rate was set at 0.8 mL/min, with UV detection at 220 nm. The injection volume was 10 μL, and samples were diluted in 50% acetonitrile to a final concentration of approximately 200 ppm. Solubility values were calculated from HPLC peak areas and expressed in milligrams per liter (mg/L). All measurements were conducted in triplicate to ensure accuracy and reproducibility.

This experiment was designed to investigate SMPTs under supersaturated or slurry conditions and does not represent a conventional dissolution study.

### 2.5. High-Resolution PXRD (HR-PXRD) and Time-Dependent HR-PXRD Study of SMPT

HR-PXRD measurements were conducted using a SmartLab diffractometer (RIGAKU Corporation, Tokyo, Japan) equipped with a D1 hybrid pixel detector in Bragg–Brentano geometry. A monochromatic Cu Kα_1_ radiation source (λ = 1.540593 Å) was used, operating at 40 kV and 50 mA. All measurements were performed at 295 ± 3 K in the continuous 2θ–θ scan mode.

For standard structural characterization, data were collected over a 2θ range of 5–70° at a scan rate of 1 °/min. These measurements were used to confirm the solid-state identity of each TPZ sample, including Polymorphs A and B and the amorphous form.

For the time-dependent study of SMPT, diffraction patterns were recorded over a narrower 2θ range of 5–35° at a scan rate of 10 °/min to monitor phase evolution over time. The samples of Polymorph B and the amorphous form were prepared as slurries and loaded onto 0.2 mm deep glass plate holders. Phase transitions were monitored at a controlled temperature of 22.0 ± 3.0 °C to assess transformation behavior under ambient laboratory conditions.

For Polymorph B, which gradually converted to Polymorph A, relative phase quantification was conducted based on two-phase composition (%). The diffraction data were analyzed using the Rietveld refinement program Profex (version 5.2) [27], which employs the BGMN refinement kernel based on a Bayesian algorithm optimized for phase quantification. The crystal structures used for Rietveld refinement were retrieved from the Cambridge Crystallographic Data Centre (CCDC 2376144 for Polymorph A and 2387615 for Polymorph B).

In the case of the amorphous form, which converted exclusively to Polymorph A, the direct quantification of the amorphous-to-crystalline ratio by Rietveld refinement was not feasible. Therefore, a calibration curve was constructed using physical mixtures of amorphous TPZ and Polymorph A in 11 known proportions (0–100% Polymorph A). These mixtures were measured as dried samples, and a standard curve was generated based on peak areas obtained from HR-PXRD data.

Although solvent-saturated conditions would more closely mimic the SMPT environment, the calibration measurements were deliberately conducted on dried powders to prevent unintended phase transformation during the calibration process, which could compromise the reliability of the standard curve.

In contrast, the actual SMPT samples were prepared as solvent-saturated slurries. To minimize solvent-induced artifacts in the diffraction profiles, the solid-to-liquid ratio was carefully controlled. Despite the hydration state differences between calibration and transformation samples, structural data were shown to be consistent and comparable across experiments.

### 2.6. DSC Analysis of SMPT

DSC measurements were performed using a TA Instruments SDT650 system (TA Instruments, New Castle, DE, USA). Approximately 3–8 mg of each sample was placed in an open alumina pan (90 μL). The samples were equilibrated at 30.0 °C and then heated from 30 °C to 300 °C at a rate of 10 °C/min under a nitrogen atmosphere (flow rate: 20 mL/min). The resulting thermal data were processed using Trios software (version 5.7.0.56; TA Instruments).

## 3. Results and Discussion

### 3.1. Summary of Key Findings

To provide a clear overview of the experimental and computational findings, this section summarizes the essential results regarding the polymorphic behavior of TPZ. The key objectives were to identify stable crystal forms, understand conformational preferences in solution, and elucidate the mechanisms governing SMPT. The most significant findings are highlighted below:

#### 3.1.1. Polymorph Identification and Thermodynamic Stability

- Three solid-state forms of TPZ were studied: Polymorph A, Polymorph B, and the amorphous form.
- Polymorph A was consistently identified as the thermodynamically stable form under all tested conditions and solvents.
- Polymorph B was observed only transiently in aprotic solvents such as acetone and MEK, indicating its metastable nature.
- The amorphous form exhibited higher solubility but converted readily to Polymorph A, especially under accelerated storage or slurry conditions.
- Solution-phase conformational preferences and tautomerism
- Conformational energy landscape mapping combined with DFT-D calculations showed that the most stable solution-phase conformers strongly resembled the structures observed in Polymorph A.
- TPZ exists as two tautomeric forms in solution. Tautomer 2 predominates in protic solvents and adopts a conformation aligned with Polymorph A.
- ROESY NMR spectra confirmed solvent-dependent NOE patterns, reflecting tautomer-specific conformational arrangements.

#### 3.1.2. Hydrogen Bonding and Crystal Packing

- DFT-D single-point energy calculations of hydrogen-bonded dimers extracted from crystal structures demonstrated that dimers in Polymorph A are energetically more favorable overall.
- Although one specific dimer motif in Polymorph B showed the lowest individual energy, it did not appear to drive stable crystallization under experimental conditions.

#### 3.1.3. Solubility and SMPT

- Solubility measurements revealed that both Polymorph B and the amorphous form gradually transformed into Polymorph A in all solvents tested (acetone, methanol, and water), confirming thermodynamic convergence.
- Time-dependent PXRD monitoring of suspensions captured the progression of SMPT from B or the amorphous form to Polymorph A. Importantly, no unknown intermediate phases or alternative crystal forms exhibiting greater stability than Polymorph A were detected under any of the tested conditions.

#### 3.1.4. Kinetic Modeling Using the KJMA Equation

- Transformation kinetics were successfully modeled using the KJMA equation, allowing the extraction of the rate constant (k) and Avrami exponent (*n*).
- Solvent-dependent differences were observed:
  ○ Methanol: fastest transformation (*k* = 0.22, *n* = 11.3 from amorphous).
  ○ Acetone: moderate kinetics with intermediate B phase (*n* = 5.5–8.4).
  ○ Water: slow transformation (*n* = 6.3–11.5), requiring DSC-based estimation.

#### 3.1.5. Mechanistic Implications and Polymorph Selection

- Solvent polarity and hydrogen-bond donor ability influenced both tautomer distribution and conformational bias, which in turn affected the polymorph outcome.
- In methanol, strong dual hydrogen bonding suppressed the formation of Polymorph B, leading directly to Polymorph A.
- In acetone, weaker solvent interactions allowed the transient stabilization of Polymorph B, which then transformed to A via a two-step pathway.
- These findings support a mechanism in which solution-phase conformers and solvent interactions collectively direct polymorph selection.
- The absence of detectable Polymorph B in protic solvents may reflect a “disappearing polymorph” scenario, a critical concern in pharmaceutical solid-state design.

### 3.2. Computational Investigation of Molecular Interactions and Stability in Solution

#### 3.2.1. Computational Analysis of Solvent-Phase Conformational Preferences

The comprehensive identification of crystal forms is essential in pharmaceutical research, as the polymorphic state of a drug can significantly affect its solubility, stability, and bioavailability. Although it is theoretically possible to explore all potential polymorphs using CSP, such efforts typically require substantial time, labor, and financial resources. Even when CSP successfully enumerates candidate structures, the number of possibilities can be overwhelming, particularly for molecules with high conformational flexibility.

**Figure 1:**
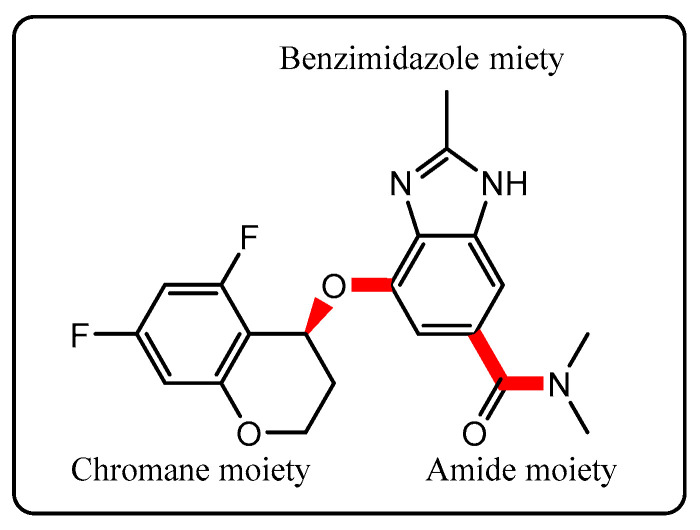
Chemical structure of TPZ ((*S*)-4-((5,7-difluorochroman-4-yl)oxy)-*N*,*N*,2-trimethyl-1*H*-benzo[d]imidazole-6-carboxamide), with four key rotatable bonds highlighted in red. These flexible sites influence their conformational and polymorphic behavior.

However, for molecules with relatively few rotatable bonds—i.e., low conformational degrees of freedom—it may be feasible to make informed predictions about plausible solid-state structures by integrating solution-phase conformational analysis, key intermolecular interaction patterns (e.g., hydrogen bonding), and known crystallographic packing motifs. This approach can narrow the scope of experimental screening, thereby improving efficiency. Moreover, it has been suggested that the most stable conformers in solution are often reflected in the structures adopted in the solid state (Figure 1).

TPZ contains four key rotatable bonds (excluding terminal methyl groups) and consists of 47 atoms, making it moderately sized yet amenable to computational investigation. These degrees of freedom are associated with two molecular regions: (i) the chromane moiety, which connects via an ether oxygen to the benzimidazole core, and (ii) the diethylamide moiety, in which the carbonyl carbon is bonded to both the benzimidazole and the amide nitrogen. In both regions, fixing one dihedral angle largely determines the other, allowing a focused scan of the dihedrals directly attached to the benzimidazole framework.

Accordingly, a relaxed coordinate scan was conducted across these key dihedral angles in 10° increments over the full 0–360° range, generating 1369 unique conformers.

**Figure 2:**
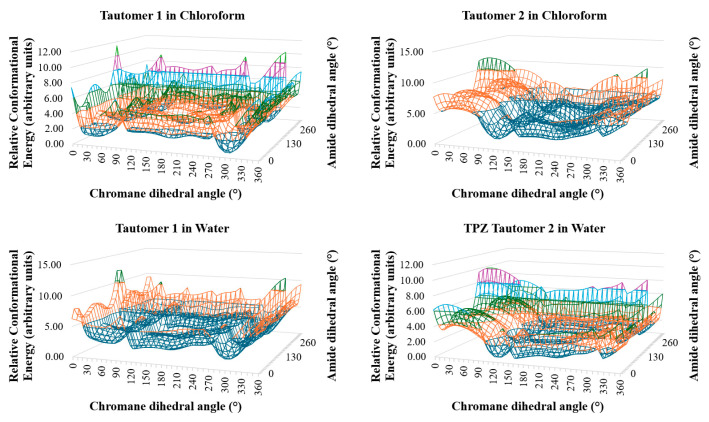
Three-dimensional conformational energy landscapes of TPZ tautomers in chloroform and water. Energy is plotted as a function of chromane and amide dihedral angles. Minima indicate probable solution-phase conformers.

**Figure 3:**
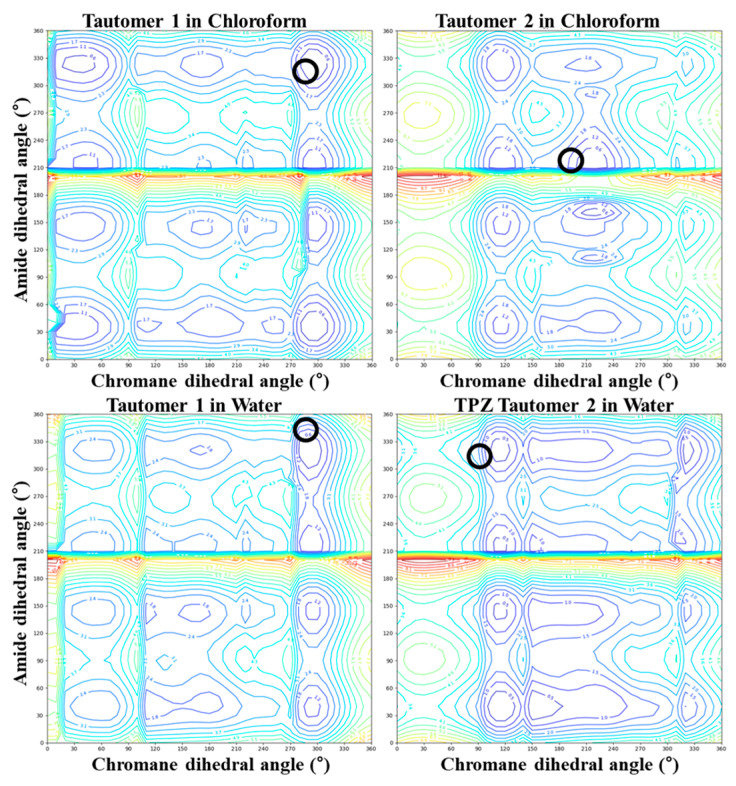
Two-dimensional contour maps of conformational energy for TPZ tautomers in chloroform and water. Black circles indicate the lowest-energy conformers.

We employed a two-tiered computational strategy: (1) an initial conformational energy mapping using the OPLS4 force field under water and chloroform solvent conditions to rapidly survey the landscape (Figure 2 and Figure 3) and (2) the subsequent refinement of selected conformers using density functional theory with dispersion corrections (DFT-D), specifically wB97X-D3(BJ)/6-31G** [28,29,30,31], under the same solvent conditions modeled using the PCM in the Jaguar module.

The force-field-based analysis enabled the identification of the global minimum conformer, which was then subjected to DFT-D geometry optimization and vibrational frequency analysis to confirm that it represented a true energy minimum (i.e., no imaginary frequencies). If imaginary frequencies were present, we expanded the search by selecting eight neighboring conformers, generated by ±10° shifts in the chromane and amide moiety dihedral angles, and we subjected them to the same DFT-D calculations to identify the most stable and physically meaningful structure.

This tiered approach was driven by the practical observation that performing DFT-D calculations for all 1369 conformers would require over a year of continuous computation on a standard personal computer, whereas the force-field-based mapping could be completed in less than a day. While DFT-D offers greater accuracy, the overall features of the conformational energy landscape can be sufficiently captured by the force-field method, making it an effective pre-screening step. Notably, TPZ exists in solutions as two tautomeric forms, and the entire computational workflow described above was applied independently to each tautomer.

**Figure 4:**
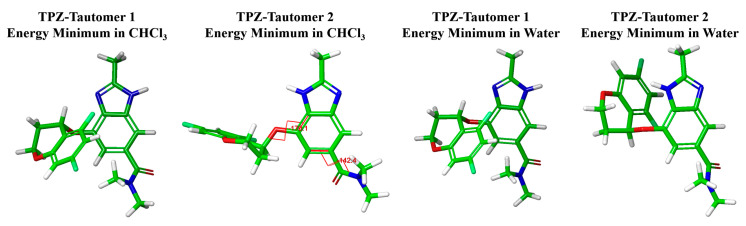
Optimized molecular geometries of the lowest-energy TPZ tautomers in chloroform and water. Atom colors: C (green), N (blue), O (red), and H (light gray).

Figure 2 and Figure 3 present the conformational energy landscapes and identify the global minima for each tautomer. Table 1 and Figure 4 summarize the corresponding dihedral angles and optimized structures of the lowest-energy conformers.

These results illustrate that even for moderately complex pharmaceutical molecules, the strategic integration of rapid force-field mapping with targeted, high-level quantum chemical refinement can efficiently and reliably identify the most stable conformers. Moreover, the observed consistency between solution-phase conformational preferences and likely solid-state arrangements underscores the relevance of such computational workflows in guiding experimental polymorph screening, thereby streamlining drug development processes.

The lowest-energy conformer of the TPZ tautomer 2, as calculated in chloroform, adopts a geometry in which the chromane moiety is positioned opposite the imidazole ring, placing the chiral center hydrogen in close proximity to the benzimidazole ring hydrogens, consistent with the NOE interactions observed experimentally. Notably, this conformer closely resembles one of the two conformers present in Polymorph A.

Conversely, the lowest-energy conformer of TPZ tautomer 1 in chloroform adopts an opposite orientation, with the chromane moiety directed toward the imidazole ring and the chiral center hydrogen positioned away from NOE-relevant spatial arrangements. While this geometry shows a general similarity in chromane orientation to the other conformer observed in Polymorph A and the two conformers identified in Polymorph B, it does not perfectly align; as reflected in Table 1, notable dihedral angle differences of approximately 20° to 90° exist between these conformers, underscoring that the correspondence is approximate rather than exact.

These observations suggest that the crystallization of TPZ proceeds preferentially from the lowest-energy solution-phase conformer of each tautomer, serving as a molecular template for the formation of specific crystal packing motifs.

At ambient temperatures (298 K), TPZ exists as a distribution of conformers in solution, with each conformer’s relative population governed by its conformational energy difference (Δ*E*) according to the Boltzmann distribution *P_i_*:

*P_i_*∝exp(−Δ*E*/(*k_B_*·T)), (1)

where Δ*E* is the energy relative to the global minimum, *k_B_* = 0.001987 kcal/mol·K is the Boltzmann constant, and T = 298 K. From this distribution, the threshold energy (Δ*E_th_*) corresponding to a cumulative probability of 99.99% is calculated to be 5.453 kcal/mol. Conformers with Δ*E* > Δ*E_th_* can be considered thermally inaccessible under ambient conditions.

The average thermal energy of a molecule at 298 K is approximately 0.6 kcal/mol, and conformational transitions with torsional barriers below 3 kcal/mol are generally considered thermally accessible. This ~3 kcal/mol threshold is widely accepted and supported by theoretical and computational studies [32,33,34].

However, it is important to note that Boltzmann-derived probabilities describe only the thermodynamic accessibility of conformers and do not necessarily reflect their kinetic behavior. Even if a high-energy conformer lies below the thermal threshold (e.g., Δ*E* ≈ 5 kcal/mol), its residence time may be extremely short due to the steep energy gradient leading toward more stable conformations. According to transition state theory, such conformers would convert rapidly to lower-energy forms, often on picosecond to nanosecond timescales. Therefore, while the energy landscape may appear broadly populated, the vast majority of molecules are effectively localized near the global minimum.

In this context, it is reasonable to conclude that the conformers most thermodynamically favored in solutions—namely, those near the global minimum—are predominantly those that are adopted during crystallization.

#### 3.2.2. Correlation Between Solution Conformers and NOE Observations

**Figure 5:**
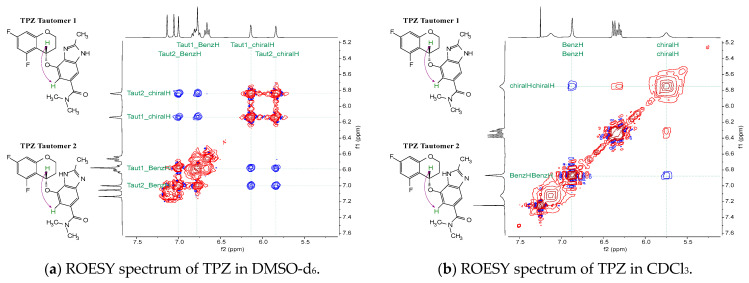
ROESY spectra of TPZ in DMSO-d_6_ and CDCl_3_. (**a**) In DMSO-d_6_, two distinct cross-peaks indicate distinct NOE interactions from both tautomeric forms. (**b**) In CDCl_3_, a single broad signal reflects rapid tautomerization and dominant conformational behavior. Schematic diagrams to the left illustrate key NOE correlations for each condition. Red contours represent positive-phase peaks and blue contours represent negative-phase peaks. The negative cross-peaks (blue) correspond to NOE signals, which are characteristic of ROESY spectra.

In solutions, TPZ exists as two tautomeric forms, tautomer 1 and tautomer 2, whereas upon crystallization, both Polymorph A and Polymorph B selectively adopt only one tautomeric form [20]. The rotating-frame Overhauser effect spectroscopy (ROESY) measurements of TPZ in CDCl_3_ and DMSO-d_6_ (Figure 5) reveal solvent-dependent spectral differences. In CDCl_3_, the spectrum displays the chiral center proton as a single broad peak, while in DMSO-d_6_, two distinct peaks corresponding to the chiral center proton are observed. This difference arises from solvent-dependent variations in the tautomerization rate [35,36].

For tautomer 1, the amide-side imidazole proton permits a molecular arrangement in which the chromane moiety is positioned toward the imidazole side. Computationally derived minimum-energy structures for tautomer 1 support this configuration, with the chromane group oriented close to the imidazole, thereby rendering the chiral center proton less accessible for observable NOE interactions.

However, in DMSO-d_6_, NOE cross-peaks between the chiral center proton and the benzimidazole ring hydrogens can be detected. The distinct chemical shifts confirm that NOE signals originate from both tautomeric forms. This reflects the slower tautomerization rate in DMSO-d_6_, allowing both forms to be detected.

Additionally, the strength of the NOE signals in DMSO-d_6_, compared to other signals, suggests that the chromane moiety spends more time positioned opposite to the imidazole side rather than remaining close to it. Interestingly, even though computational models predict that, in water or chloroform, the most stable conformer of tautomer 1 places the chromane moiety near the imidazole—where NOE interactions would not be expected—the ROESY spectra suggest that, in reality, the system predominantly adopts the lowest-energy conformer of tautomer 2 under chloroform conditions.

The reason for this lies in the structural features of tautomer 2: The presence of an imidazole-side hydrogen introduces steric hindrance, forcing the chromane moiety to adopt the opposite orientation in its lowest-energy conformation. Because the tautomeric hydrogen exchange occurs rapidly, even if the system transiently shifts to tautomer 1, it quickly reverts to tautomer 2, thereby maintaining the chromane moiety on the opposite side. Consequently, the molecule—even when formally present as tautomer 1—is effectively constrained to remain in a conformation resembling the lowest-energy form of tautomer 2.

#### 3.2.3. Hydrogen Bonding Mechanisms in Crystallization Guided by Solution Conformers

In Section 3.2.1, we identified the most stable solution-phase conformers through computational methods, and in Section 3.2.2, we predicted their spatial arrangements in solution using NOE spectra. Building on these results, we sought to infer how these conformational preferences influence the crystallization process and to what extent they allow the predictive assessment of polymorph formation.

It is widely suggested that crystallization often proceeds from the most stable solution-phase conformer, which can efficiently pack into an energetically favorable and stable crystal lattice [5,37]. For neutral organic molecules, hydrogen bonding generally represents the strongest and most directional intermolecular interaction, playing a central role in guiding the early stages of crystal assembly [38]. Once hydrogen-bonded dimers are formed, additional non-covalent interactions, such as π–π stacking and π–H interactions, further stabilize the resulting crystal packing motif.

To investigate how hydrogen bonding patterns govern the crystallization of TPZ, we extracted representative hydrogen-bonded dimers from the crystal structures of Polymorph A and Polymorph B and performed single-point energy calculations to compare their relative stabilities. These calculations provide insights not only into the energetic differences between the polymorphs but also into the likely hydrogen bonding scenarios operating during solution-phase preorganization and nucleation.

For these calculations, we used density functional theory with dispersion corrections (DFT-D), specifically the wB97X-D3(BJ)/def2-TZVPP level of theory, applying a PCM to simulate chloroform as the solvent. We intentionally avoided the geometrical optimization of the extracted dimers prior to the single-point calculations because preliminary tests showed that optimization frequently led to significant structural distortions, deviating considerably from the original crystal configurations. Additionally, we opted for the def2-TZVPP basis set instead of the smaller 6-31 basis set** to better capture long-range interactions and improve calculation accuracy. Although we attempted to further refine the energy calculations using the CCSD(T)/def2-TZVPP method [39,40], the system’s size (94 atoms) exceeded available PC resources, resulting in excessive input/output demands and rendering such high-level calculations computationally infeasible.

**Figure 6:**
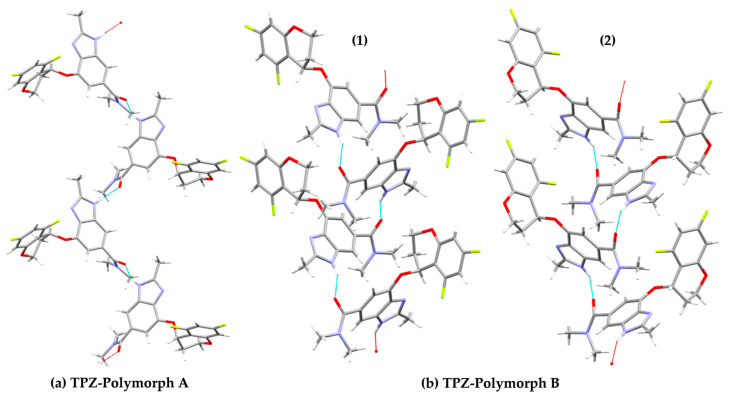
Hydrogen-bonded molecular arrangements in the crystal structures of TPZ Polymorphs A and B. (**a**) Polymorph A shows alternating conformers forming two distinct hydrogen bonds within the asymmetric unit (Z′ = 2). (**b**) Polymorph B exhibits two types of hydrogen-bonded chains labeled (1) and (2). Dashed lines indicate hydrogen bonds. Atom colors: carbon (gray), nitrogen (blue), oxygen (red), fluorine (yellow green), and hydrogen (white).

In Polymorph A, the crystal structure features two types of hydrogen bonds: one between the imidazole hydrogen and the amide carbonyl oxygen, alternating between two distinct conformers due to the asymmetric unit (Z′ = 2) (Figure 6a) [20]. In contrast, Polymorph B contains repeating chains of identical conformers, stabilized by hydrogen bonding and π–π stacking interactions (Figure 6b). Accordingly, we calculated the total energy of two hydrogen-bonded dimer pairs in Polymorph A and two analogous dimers in Polymorph B. For Polymorph B, both dimer types were formed by identical conformers engaging in hydrogen bonding.

The results revealed an intra-polymorph energy difference of 0.89 kcal/mol between the two hydrogen-bonded dimer types in Polymorph A, indicating minor variations in hydrogen bond stability within the same crystal form. In contrast, Polymorph B showed a much larger energy difference of 12.44 kcal/mol between its two hydrogen-bonded dimer types. When comparing the average hydrogen-bonded dimer energies across polymorphs, Polymorph A was found to be more stable overall, with an average energy 3.29 kcal/mol lower than that of Polymorph B. Notably, however, one specific hydrogen-bonded dimer in Polymorph B exhibited an energy approximately 2.5–3.4 kcal/mol lower than either of the dimers in Polymorph A (Table 2).

This intriguing finding raises the question of whether, under certain conditions, the most stable hydrogen-bonded dimer motif—if it could exist independently—might enable the formation of a previously unknown, more stable crystal structure. To test this hypothesis, long-term solubility studies or time-dependent phase transformation experiments would be required to determine whether such a transformation could be induced or observed experimentally.

Moreover, the preference for tautomer 1 in hydrogen-bond-driven crystallization can be rationalized by considering steric effects: In tautomer 2, the chromane-side hydrogen introduces steric hindrance, preventing close approach by neighboring molecules and thereby inhibiting effective hydrogen bonding. In contrast, tautomer 1, with its amide-side hydrogen, presents a less sterically hindered environment, which facilitates molecular alignment and promotes hydrogen bond formation.

### 3.3. Solubility Measurements and Thermodynamic Stability of Polymorphs

Building on previous findings that solution-phase conformers resemble those in crystal structures, we hypothesized that crystallization is driven by these conformational preferences. The computational results further suggested that Polymorph A is stabilized by favorable hydrogen-bonding networks. However, as shown in Table 2, a specific conformer of Polymorph B forms a hydrogen-bonded array with the lowest calculated energy, raising the concern that this packing arrangement could also potentially occur.

To comprehensively assess the crystallization behavior, we included not only Polymorphs A and B but also the amorphous form in the solubility measurements. The amorphous form, while enhancing solubility, may gradually convert to crystalline forms, affecting stability. Solubility tests were performed at 20.0 ± 0.5 °C over 48 h in three solvents: methanol (a protic solvent), acetone (an aprotic solvent), and water (a polar solvent expected to have the most significant impact on the drug). The concentration of TPZ in solution was quantified by HPLC. Measured solubility values are summarized in Table 3.

**Figure 7:**
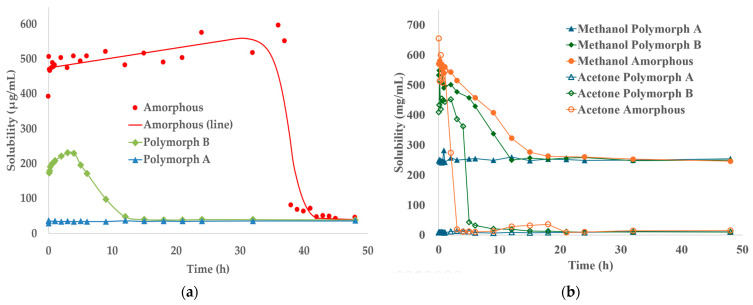
Time-dependent solubility profiles of TPZ Polymorphs A and B and the amorphous form under supersaturated conditions at 20.0 ± 0.5 °C. Subfigure (**a**) shows solubility changes in water (μg/mL), while (**b**) presents solubility behavior in methanol and acetone (mg/mL). In all solvents, the solubility of each form gradually converged toward that of Polymorph A, indicating its thermodynamic stability.

The experimental results are presented in Figure 7, which illustrates the solubility profiles of Polymorphs A and B and the amorphous form of TPZ in the three tested solvents—methanol, acetone, and water. Although the initial solubility values exhibited minor fluctuations due to the nonequilibrium nature of the supersaturated system, the overall solubility trends were clear and consistent. Specifically, both Polymorph B and the amorphous form showed a gradual decrease in solubility over time, ultimately converging toward the equilibrium solubility of Polymorph A. This behavior was observed in all three solvents, underscoring the thermodynamic stability of Polymorph A under the tested conditions. These results highlight the solvent-driven transformation toward thermodynamic equilibrium rather than absolute solubility differences.

The observation that the solubility profiles of both Polymorph B and the amorphous form converge toward that of Polymorph A strongly suggests that, under the experimental temperature and pressure conditions employed in this study, the formation of alternative, potentially more stable crystalline forms is not favored. Although certain Polymorph B conformers exhibited lower-energy hydrogen-bonded motifs in silico, these did not manifest experimentally under the tested conditions. This finding reinforces the robustness of Polymorph A as the thermodynamically stable form under ambient conditions, highlighting its relevance as the primary crystallization product during SMPTs.

### 3.4. Phase Transformation Monitoring Under Suspension Conditions by HR-PXRD

PXRD analyses under accelerated storage conditions (40 °C/75% RH) revealed that both the amorphous form and Polymorph B convert to Polymorph A within eight weeks (Section S1), whereas no significant changes were observed by DSC under ambient or refrigerated conditions.

To determine whether this transformation proceeds via melting or is mediated by solvent, DSC was performed. DSC revealed endothermic events at approximately 100 °C for the amorphous form and ~167 °C for Polymorph B, corresponding to thermal transitions including melting. However, no distinct glass transition or thermal event associated with the transformation into Polymorph A was observed, indicating that the phase transitions discussed in this study occur below the melting point and do not involve either melting or glass transition.

The computational analyses and solubility convergence data presented in the preceding sections have been interpreted from the perspective of crystallization behavior. While the conversion from the amorphous form to Polymorph A may reasonably be considered a crystallization process, the transformation from Polymorph B to Polymorph A involves a transition between two distinct crystalline phases and may therefore proceed via a different mechanism. One possible mechanism for such transitions is SMPT, whereby the system gradually approaches the thermodynamically stable phase through solution-mediated dissolution and recrystallization. Solubility convergence serves as an indirect indicator of SMPT, while time-dependent PXRD directly monitors solid-phase changes.

SMPT is generally driven by differences in thermodynamic stability between polymorphs and may have important implications for the performance and long-term stability of pharmaceutical products. The computational analysis described in Section 3.2.3 suggested that locally favorable, low-energy packing motifs associated with Polymorph B might transiently form under certain conditions. However, experimental solubility measurements indicated that both the amorphous form and Polymorph B ultimately converge to the solubility profile of Polymorph A, supporting its thermodynamic stability. Nevertheless, solubility data alone cannot fully elucidate the presence of intermediate phases or the precise transformation pathways. Therefore, in the present study, the time-dependent PXRD measurements of suspensions were employed to directly track the evolution of solid-state composition, providing further insight into the kinetic pathways leading toward thermodynamic equilibrium.

From a mechanistic standpoint, the transformation from Polymorph B to Polymorph A may be regarded as a solid-to-solid crystalline phase transition, whereas the amorphous-to-Polymorph A conversion represents nucleation and the growth of a crystalline phase. Although both pathways ultimately yield the thermodynamically stable Polymorph A, they likely proceed via distinct routes, influenced by the physical characteristics of the initial phase. Thus, while recognizing these mechanistic distinctions, both processes can be reasonably discussed within the broader framework of solvent-mediated transformations.

HR-PXRD measurements for SMPT analysis were conducted over a 2θ range of 5° to 35°. For time-resolved PXRD, the 2θ range was limited to 5–35°, which captured approximately 90% of the total diffraction intensity for both polymorphs, validating its use for monitoring SMPT progression (Figure S5). Therefore, this limited range was considered sufficient for tracking phase transformation trends during SMPT.

#### 3.4.1. Time-Dependent HR-PXRD Analysis of Polymorph B Suspension

To further investigate the SMPT behavior of TPZ, time-dependent PXRD measurements were conducted using three representative solvents with distinct polarities: methanol, acetone, and water. The transformation profiles to the thermodynamically stable Polymorph A were quantitatively analyzed over time and fitted using the KJMA equation to model the kinetics. The kinetic parameters derived from these fits, including the rate constant (*k*) and Avrami exponent (*n*), are summarized in Table 4. It should be noted that phase fractions were estimated by Rietveld refinement and represent relative and not absolute quantities of each polymorph in the measured samples. The refinement quality was acceptable (Rwp ~9%, Rexp ~5%, χ^2^ < 5), meeting commonly used criteria for PXRD analysis [41,42,43].

Among various solid-state kinetic models discussed in the literature [25,44], the KJMA equation was selected based on its applicability to nucleation-growth processes and its consistency with the transformation profiles observed in this study. The selection was further supported by comparative criteria outlined by Khawam and Flanagan, who extensively reviewed model applicability depending on the shape of fractional conversion curves [25]. Its applicability to solid-state SMPT systems has been widely recognized. The general form of the equation is given as follows:

*Y*(*t*) = 1 − exp(−*k*⋅*t^n^*), (2)

where *Y*(*t*) represents the transformed fraction at time *t*, *k* is the apparent rate constant, and *n* is the Avrami exponent, which reflects the dimensionality and mechanism of the transformation. This model allows the empirical fitting of experimental data to extract kinetic parameters characterizing the transformation process.

**Figure 8:**
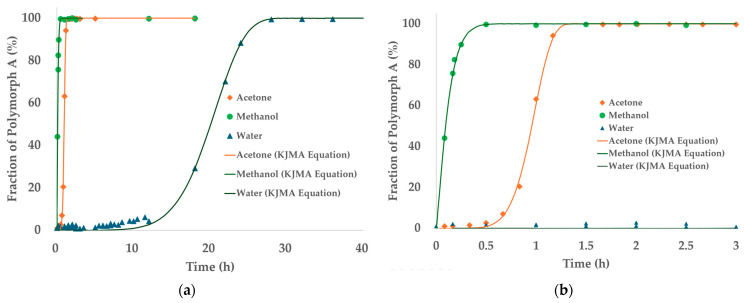
PXRD-based phase transformation profiles of the TPZ Polymorph B to Polymorph A transformation in different solvents, with KJMA model fits. (**a**) Full profile (0–40 h) and (**b**) enlarged initial phase (0–3 h).

Figure 8 presents the time-dependent transformation profiles of TPZ Polymorph B into Polymorph A for the three solvents studied—acetone, methanol, and water. In panel (a), the full transformation profiles over a 40 h period are shown, with experimental data points superimposed with fitted curves based on the KJMA model. The transformation rates varied substantially depending on the solvent: rapid conversion was observed in methanol and acetone, while water displayed a much slower transformation with an extended induction period.

Panel (b) offers a magnified view of the initial 3 h period to emphasize differences in early-stage kinetics among the solvents. Notably, methanol exhibited extremely rapid transformation, achieving nearly complete conversion within 1 h. In contrast, acetone showed slightly slower kinetics with a longer induction period, while water required over 20 h to reach comparable levels of transformation.

These results confirm solvent-dependent SMPT behavior and support the kinetic analysis using the KJMA model. Although only methanol, acetone, and water are discussed in the main text to highlight contrasting transformation behaviors, additional data for 2-propanol and toluene—which exhibit intermediate transformation profiles between acetone and water—are provided in the Supplementary Materials (Section S3, Table S4 and Figure S10). The inclusion of these solvents further supports the observed trend and offers a broader comparison across a wider polarity range.

Here, we sought to correlate the experimental phase transformation kinetics observed by PXRD with the underlying molecular and energetic features elucidated through computational conformational analysis and solubility studies. The transformation to Polymorph A observed across all solvents is consistent with predicted solution-phase conformational preferences, which favor geometries corresponding to the crystal packing motif of Polymorph A.

Furthermore, our DFT-D energy calculations of hydrogen-bonded dimers indicated that the molecular interactions stabilizing Polymorph A are energetically more favorable than those in Polymorph B, despite the presence of a particularly stable motif in one of the B dimers. These computational findings align with the solubility convergence experiments, in which both the amorphous form and Polymorph B exhibited time-dependent solubility profiles converging toward that of Polymorph A, strongly supporting the occurrence of SMPT.

The kinetic behavior revealed by time-dependent PXRD, particularly the solvent-dependent differences in induction periods and transformation rates, further substantiates this SMPT mechanism. Importantly, throughout the PXRD monitoring experiments, no additional diffraction peaks were observed that could indicate the formation of unidentified crystalline phases. All measured patterns were successfully fitted using Rietveld refinement models based on Polymorphs A and B, with no unexplained residual peaks, confirming that the transformation proceeds exclusively toward the known stable form.

Taken together, the integration of molecular-level modeling, energetic analysis, and experimental phase monitoring provides a coherent and predictive framework for understanding the polymorphic behavior of TPZ. This multidisciplinary approach strengthens the conclusion that Polymorph A is not only the thermodynamic endpoint but also the kinetically favored product under pharmaceutically relevant conditions.

As shown in Table 4, significant solvent-dependent variations were observed in both the rate constant (*k*) and the Avrami exponent (*n*), reflecting differences in nucleation and growth mechanisms. The Avrami exponent provides insight into the transformation pathway: *n* ≈ 1 typically corresponds to one-dimensional growth with instantaneous nucleation; *n* ≈ 2–3 suggests two- or three-dimensional growth with either instantaneous or progressive nucleation; *n* ≥4 implies complex mechanisms involving nucleation and impingement effects [25,45,46,47].

Methanol displayed the highest transformation rate, with an *n* value near 1.24, suggesting one-dimensional growth with instantaneous nucleation, while acetone exhibited slightly slower kinetics. In contrast, water showed a markedly lower rate constant and an *n* value indicative of progressive nucleation and three-dimensional growth. These differences underscore the critical role of solvent properties in governing SMPT behavior.

The rate constant *k* depends on the unit of time (here, hours), and thus, comparisons across studies should ensure unit consistency.

#### 3.4.2. Time-Dependent HR-PXRD Analysis of Amorphous Suspension

In the SMPT experiments initiated from the amorphous form, only the formation of Polymorph A was anticipated. Therefore, monitoring the transformation progress by comparing relative phase fractions via Rietveld refinement was deemed impractical, as no distinguishable peaks from intermediate crystalline phases were expected.

To enable the quantitative analysis of crystallinity during the SMPT process, binary physical mixtures of amorphous TPZ and Polymorph A were prepared in 10% increments, ranging from 0% to 100% Polymorph A by weight. These calibration samples were used to construct a standard curve specific to each experimental run. To minimize measurement variability due to instrumental and environmental factors, all calibration measurements were performed under the same conditions as the corresponding SMPT experiments (Section S2).

Since slurry and dried samples can differ in diffraction behavior due to solvent effects, we minimized residual solvent content by carefully controlling the solid-to-liquid ratio in the slurry samples. This procedure was applied to both methanol and acetone systems.

The calibration curve was modeled using a second-order polynomial regression to account for nonlinearities in diffraction response. The fitted curve exhibited excellent agreement with the measured data, as indicated by narrow 95% confidence and prediction intervals (see Supplementary Materials, Section S2). This confirmed the robustness of the calibration method for quantifying crystallinity during SMPT.

This nonlinearity likely arises from a combination of factors, including preferred orientation effects, variations in packing density, changes in crystallite volume fraction, and combined peak summation employed to mitigate orientation bias [48]. Collectively, these factors justify the use of a quadratic regression model to better capture the physical behavior of the PXRD signal across the full crystallinity range.

Measurement conditions for the time-dependent HR-PXRD experiments were as follows: Suspensions of amorphous TPZ in methanol, acetone, and water were monitored under continuous stirring. Diffraction data were collected at 5 min intervals during the initial stages of transformation (typically 30–60 min depending on the solvent), with total measurement times adjusted based on the transformation rate for each solvent system.

**Figure 9:**
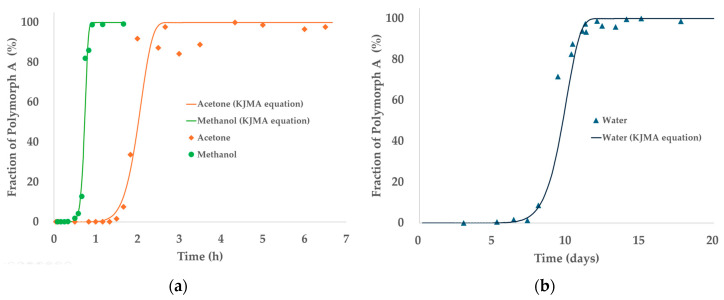
Fractional crystallinity over time in three solvent systems: (**a**) acetone and methanol and (**b**) water. Symbols represent experimental data, and solid lines represent model fits using the KJMA equation. The *x*-axis time scales differ due to variations in transformation rates among solvents.

Time-dependent PXRD data were analyzed using the KJMA equation to model crystallization kinetics from the amorphous state in acetone, methanol, and water. As shown in Figure 9, rapid phase transformation was observed in methanol and acetone, reaching over 90% crystallinity within a few hours, whereas crystallization in water proceeded significantly more slowly, requiring several days to approach completion.

For methanol and acetone, the KJMA model successfully fitted the PXRD-derived crystallinity profiles. In contrast, no detectable crystallization was observed in water during HR-PXRD monitoring, and the transformation profile was instead estimated from DSC measurements (Table S2) under controlled heating conditions. Due to the much slower crystallization kinetics in water, the *x*-axis time scale differs from that used for methanol and acetone in Figure 9.

These solvent-dependent differences in kinetic behavior likely reflect variations in solubility, molecular mobility, and supersaturation levels, all of which influence nucleation and growth dynamics. Notably, the excellent agreement between experimental data and the KJMA model across all solvent systems supports the appropriateness of this kinetic framework for describing the transformation process.

The extended time scale observed in water further reinforces the need to consider time-dependent solvent effects when designing crystallization processes, particularly in systems prone to solid-state transformations. This underscores the importance of kinetic modeling not only for mechanistic understanding but also for practical process optimization.

The kinetic parameters derived from the KJMA model are summarized in Table 5. Both the rate constant (*k*) and Avrami exponent (*n*) varied considerably among solvents. Methanol showed the fastest transformation (*k* = 0.22, *n* = 11.3), followed by acetone (*k* = 1.84 × 10^−3^, *n* = 8.42), while water exhibited an extremely low rate constant (*k* = 3.94 × 10^−28^), indicating markedly slower kinetics.

All systems yielded Avrami exponents greater than 8, suggesting highly cooperative transformations involving both nucleation and growth, likely under diffusion-controlled regimes. These values also reflect the complex crystallization geometry of amorphous systems during SMPT.

These results reinforce the conclusion that the solvent environment plays a critical role in governing transformation kinetics. The quantitative fitting provided by the KJMA model not only captures time-dependent crystallinity evolution but also enables the mechanistic interpretation of the transformation pathway, offering valuable guidance for solvent selection in pharmaceutical solid-state design.

In the case of water, no transformation was detected by PXRD even after one week. Therefore, DSC was employed to estimate the crystallization extent based on the enthalpy of melting of Polymorph A. It is worth noting that these measurements were conducted using a combined DSC–TGA instrument, rather than a dedicated high-precision DSC device. While the absolute accuracy may be somewhat limited compared to specialized equipment, the results were sufficiently consistent to capture overall trends in crystallinity development.

To address the time-dependent limitation inherent to DSC, a set of parallel samples was prepared with staggered initiation times, allowing each to be analyzed at a predefined interval. A melting endotherm corresponding to Polymorph A was consistently observed around 225 °C. Because the samples contained residual water, the actual dry weight was determined from the stable plateau region of the TGA curve between 120 °C and 180 °C, following complete dehydration. The enthalpy associated with the melting event was then normalized to the corrected dry mass, enabling an approximate yet informative estimation of the crystalline fraction. These values were used to construct the kinetic profile shown in Figure 9b.

While this methodology was adopted as a practical workaround to address experimental constraints, the resulting profiles were reasonably consistent with expectations and aligned with previously reported semi-quantitative applications of DSC for estimating low levels of crystallinity [49].

#### 3.4.3. Solvent-Directed Tautomerization and Its Influence on Polymorph Selection

**Figure 10:**
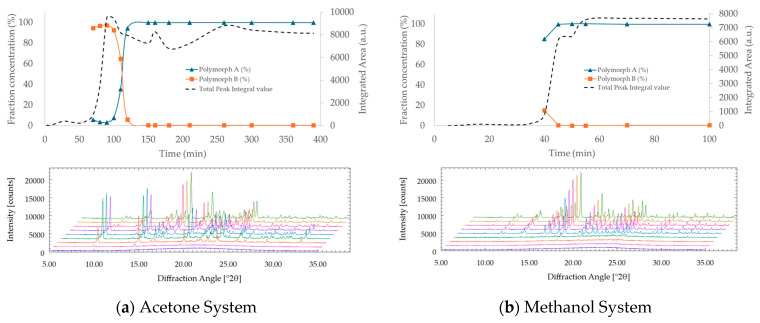
PXRD-based analysis of polymorphic transformation and crystallinity development of TPZ during SMPT in (**a**) acetone and (**b**) methanol. The upper panels show phase fractions of Polymorphs A and B (left axis) and total integrated peak area (right axis) from Rietveld refinement. The lower panels present the corresponding time-dependent PXRD patterns. In acetone, Polymorph B appears transiently before conversion to Polymorph A, whereas in methanol, Polymorph A forms directly without detectable intermediates.

TPZ exhibited distinct SMPT pathways and kinetics depending on the solvent environment. In methanol, a strongly protic solvent, Polymorph A formed directly without any detectable intermediate phases. In contrast, in acetone, Polymorph B transiently appeared and gradually transformed into Polymorph A, in line with Ostwald’s Rule of Stages (Figure 10). A similar two-step B → A transformation was also observed in MEK (Figures S11 and S12), further highlighting the solvent-dependent nature of the polymorphic transition.

In methanol, Polymorph A emerged within ~60 min, accompanied by a concurrent increase in PXRD intensity, suggesting simultaneous nucleation and growth. Despite methanol’s ~26-fold higher solubility compared to acetone, no reversion to amorphous or alternative phases was observed, indicating the stabilizing role of protic hydrogen bonding. In acetone, at 40 min, Rietveld refinement estimated approximately 20% of Polymorph B, although its peak intensity was <8% relative to Polymorph A at 100 min. This transient presence, though visually subtle, confirms a metastable intermediate phase that precedes the formation of the stable polymorph.

**Figure 11:**
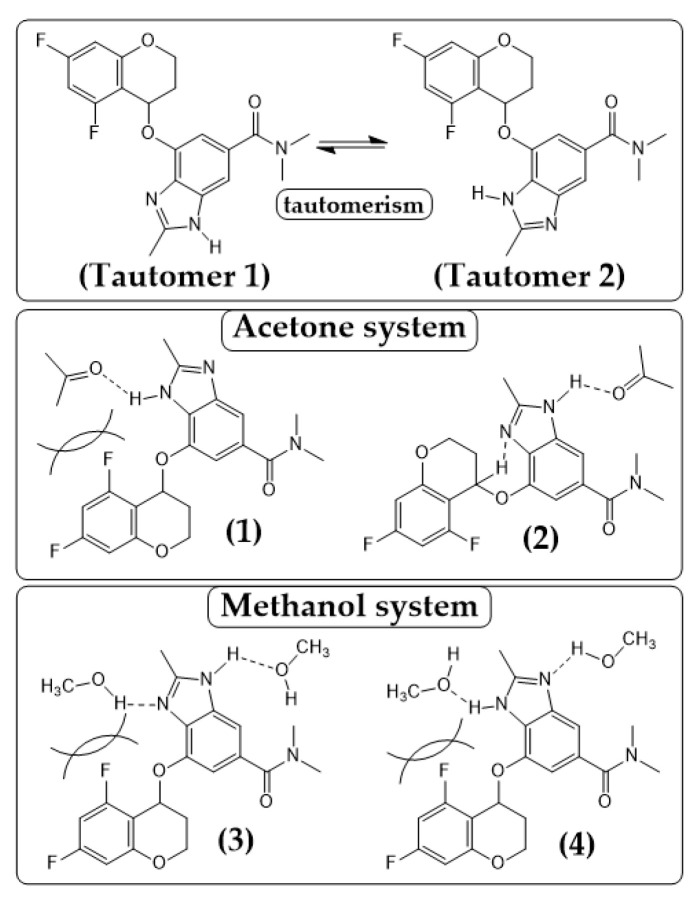
Tautomeric forms and representative hydrogen-bonded conformers of TPZ in acetone and methanol. The top panel shows Tautomer 1 and Tautomer 2, which differ by a proton shift within the benzimidazole moiety. The middle panel illustrates acetone-stabilized conformers involving weak hydrogen bonds: in conformer (1), acetone interacts near the chromane site, leading to steric hindrance, whereas in conformer (2), acetone is positioned at a site with less steric congestion, where the chiral hydrogen of the chromane moiety forms a weak intramolecular hydrogen bond with the imidazole nitrogen. The bottom panel presents methanol-stabilized conformers featuring dual hydrogen bonds: in both (3) and (4), methanol molecules are located on either side of the TPZ core, but the strength of the hydrogen bonds is asymmetrically distributed. These solvent-specific interactions significantly influence tautomeric distribution and polymorph selection during crystallization.

The difference between acetone and methanol systems is likely rooted in hydrogen-bonding interactions (Figure 11). In aprotic solvents like acetone, steric hindrance around the chromane moiety limits intramolecular hydrogen bonding, thereby increasing conformational flexibility and facilitating the formation of Polymorph B-like structures. In particular, the weak interaction between the chromane proton and the imidazole nitrogen (D···A ≈ 3.35 Å, angle ≈ 118°) may contribute to stabilization [50,51].

In contrast, methanol forms dual hydrogen bonds with both nitrogen atoms of the benzimidazole ring, altering the tautomeric equilibrium and suppressing B-like conformers. This promotes a conformation favorable to Polymorph A (second from the left in Figure 4), accelerating nucleation and growth. Once a Polymorph A-like structure forms in a solution, it may act as a seed and guide crystallization cooperatively.

We hypothesize that the polymorph selectivity arises from such solvent-induced tautomeric interconversion. As shown in Figure 11(1),(2), acetone interacts weakly with the N–H group of the benzimidazole moiety, while methanol forms stronger, dual hydrogen bonds (Figure 11(3),(4)) [52,53,54]. According to the hydrogen bond donor/acceptor hierarchy [38], hydroxyl groups (methanol) act as stronger donors than carbonyl groups (acetone), thus requiring more energy for desolvation and extending the solubility equilibration time.

**Figure 12:**
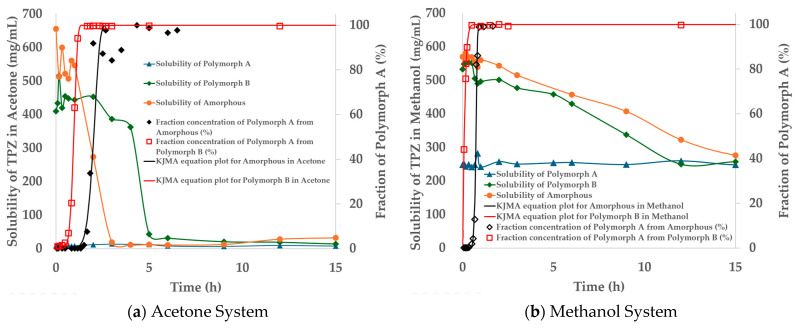
Solubility profiles and SMPT kinetics of TPZ in (**a**) acetone and (**b**) methanol. Left axes show solubility (mg/mL) of amorphous, Polymorph A, and Polymorph B; right axes show fractional conversion to Polymorph A from either amorphous (black diamonds) or Polymorph B (red squares). Solid lines indicate KJMA model fitting.

Indeed, as shown in Figure 12, SMPT from amorphous to Polymorph A in acetone took ~3 h, while B → A occurred faster (~1.5 h), though solubility stabilized only after ~5 h. In methanol, SMPT to Polymorph A was completed within 1 h (from amorphous) and 0.5 h (from B), but solubility equilibration required ~15 h, suggesting that desolvation is the rate-limiting step [44,55,56].

Despite this kinetic barrier, SMPT in methanol bypassed Polymorph B entirely, proceeding directly to the stable form. In contrast, the two-step transformation in acetone implies a structurally mediated pathway. Polymorph B likely acts as a pre-organized scaffold that reduces the nucleation barrier for Polymorph A [36,37]. This partially ordered intermediate facilitates faster conversion than direct crystallization from amorphous TPZ, which lacks structural templates and demands greater molecular reorganization.

These results clearly demonstrate that solvent selection modulates polymorphic outcomes through its effects on tautomeric equilibrium, conformational preference, and desolvation kinetics. Notably, the absence of detectable Polymorph B in methanol may resemble a “disappearing polymorph” phenomenon, as the metastable form becomes unobservable under such conditions. However, its reproducible appearance in aprotic solvents suggests that appropriate crystallization control may enable selective isolation.

Under all solution-mediated conditions tested—including accelerated storage (40 °C/75% RH) and slurry experiments—no transformation from Polymorph A to other forms was observed, confirming its thermodynamic stability. Nonetheless, external mechanical stress (e.g., milling, compression) was not evaluated and may warrant future investigation, as such factors can induce amorphization in other APIs.

### 3.5. Broader Context and Future Perspectives

To contextualize the polymorphic behavior of TPZ within the broader class of P-CABs and identify future research directions, this section presents a comparative analysis with other P-CABs and outlines the key limitations and opportunities for advancing polymorph prediction and control in tautomeric drug compounds.

#### 3.5.1. Structural Comparison with Other P-CABs and Mechanistic Implications

Several P-CABs—including vonoprazan, revaprazan, and soraprazan—have been studied for their physicochemical and solid-state properties [57,58,59,60,61,62,63,64]. Unlike TPZ, these compounds lack tautomeric behavior, as their core heterocyclic moieties do not support intramolecular proton transfer. Consequently, their solid forms are primarily governed by salt formation, hydration, or packing effects rather than conformational interconversion.

In contrast, TPZ undergoes tautomerism within the benzimidazole ring, resulting in significant conformational shifts that influence hydrogen-bonding potential and polymorphic outcomes. As shown in this study, solvent-dependent tautomer distribution directly impacts the likelihood of forming either Polymorph A or B during crystallization.

This tautomerism-driven complexity distinguishes TPZ from other P-CABs and may underlie phenomena such as the following:

- Solvent-mediated phase transitions (SMPTs) with transient intermediate states.
- Disappearing polymorphs, particularly Polymorph B, under protic solvent conditions.
- Broader conformational landscapes, increasing the unpredictability of crystallization.

These differences suggest that tautomerism introduces an additional structural degree of freedom, increasing the risk of uncontrolled polymorphic behavior. Therefore, TPZ serves as a model for tautomer-sensitive crystallization while highlighting the need for tailored polymorph screening strategies beyond conventional methods applied to non-tautomeric APIs.

#### 3.5.2. Study Limitations and Future Directions

##### Study Limitations

1. The conformational and hydrogen-bonding analyses were conducted only for selected solvents (chloroform and water), while methanol and acetone—despite their demonstrated experimental significance—were not explicitly included in DFT-D modeling. This limitation arose due to restrictions in the solvent models available within the force field and PCM settings of the software used (OPLS4 and Jaguar), which do not provide comprehensive parameterization for all solvents at the quantum mechanical level.
2. Hydrogen bonding was analyzed at the dimer level without incorporating periodic boundary conditions or full lattice energy calculations, limiting direct comparisons of overall crystal packing stability. This was primarily due to computational resource limitations, as periodic DFT-D calculations for complex organic crystals—particularly with a large asymmetric unit (Z′ = 2)—require substantial memory and CPU time that exceeded the available system capabilities.
3. The tautomeric equilibrium was inferred primarily from NOE-based NMR and supported by solution-phase modeling, but the quantitative measurement of tautomer populations in various solvents was not performed.
4. Comparative analyses with other P-CABs were limited to structural features; the polymorphic behaviors of vonoprazan, revaprazan, and soraprazan were not studied experimentally under analogous crystallization conditions.

##### Future Directions

1. Extend DFT-D conformational and hydrogen-bonding analyses to additional solvents (e.g., methanol, acetone) as software capabilities evolve to quantitatively link solvent effects with polymorph selectivity.
2. Perform full periodic DFT optimizations and lattice energy calculations for each polymorph, once sufficient computational resources are available, to rigorously assess thermodynamic stability and crystal packing forces. These high-level calculations will also enable comparative evaluation against current lower-cost approaches—such as dimer-based DFT-D and force-field-based methods—to determine whether such approximations can reliably capture relative polymorph stability. Establishing this correlation would validate the continued use of computationally efficient methods in early-stage polymorph screening, especially for compounds with large unit cells or limited resources.
3. Expand the comparative study to other P-CABs (e.g., vonoprazan, revaprazan, soraprazan) by applying the same analytical workflow. Although these compounds do not exhibit tautomerization, parallel studies could reveal whether other subtle conformational or hydrogen-bonding effects influence polymorph selection and transformation kinetics in this drug class.
4. More broadly, the role of tautomerism in polymorphic diversity warrants systematic investigation across tautomeric APIs, with TPZ serving as a mechanistic model for tautomer-driven crystallization.

## 4. Conclusions

In this study, we investigated the polymorphic behavior of TPZ using a combined computational and experimental approach. DFT-D analysis revealed that the most stable solution-phase conformers—particularly in chloroform—closely resemble those observed in Polymorph A. This prediction was experimentally supported by NOE-based NMR, underscoring the importance of hydrogen bonding and tautomerism in directing polymorphic outcomes.

Time-dependent PXRD and solubility studies demonstrated solvent-mediated phase transitions (SMPTs). In aprotic solvents like acetone, metastable Polymorph B formed transiently and subsequently transformed into Polymorph A, consistent with Ostwald’s Rule of Stages. In contrast, protic solvents such as methanol directly induced Polymorph A without detectable intermediates. Slurry systems exhibited faster transformations, suggesting that ambient moisture or residual solvents may accelerate phase changes during storage.

Although DSC showed no significant changes after three months at 5 ± 3 °C or 25 ± 2 °C/60 ± 5% RH, time-dependent PXRD and solubility data indicate that slow transitions can occur even near room temperature, underscoring the importance of long-term stability monitoring and conservative storage.

Tautomerism within the benzimidazole ring modulates conformational equilibrium, hydrogen bonding, and crystallization pathways. Kinetic modeling revealed distinct mechanisms for amorphous-to-A and B-to-A transitions, reflected in high (*n* > 8) versus moderate (*n* = 1–6) Avrami exponents, respectively.

These findings support a CSP-independent, mechanism-based strategy for polymorph selection, grounded in solution-phase behavior and solute–solvent interactions, and potentially applicable to other tautomeric APIs.

From a formulation perspective, oral administration remains the preferred route for TPZ, as reflected in its current clinical use. Although all polymorphic forms are readily soluble in organic solvents, their poor aqueous solubility presents formulation challenges for both oral and parenteral delivery. For oral dosage forms, enhancing bioavailability through solid dispersions or amorphous formulations may be warranted. However, the superior stability of Polymorph A and its dominance under physiological conditions make it the most suitable choice for long-term oral administration. Parenteral formulations were not explored in this study but would require solubilization strategies due to the limited water solubility.

Given its superior thermodynamic and kinetic stability, Polymorph A—already used in marketed TPZ products—should remain the target form in pharmaceutical development. To avoid the formation of the metastable Polymorph B, aprotic solvents such as acetone and MEK should be excluded from crystallization and formulation. Instead, protic solvents like methanol or ethanol are preferred. As phase transitions can occur even at ~20 °C over time, long-term storage under refrigerated conditions (≤5 °C) is recommended. This mechanism-informed strategy ensures solid-state consistency and enhances product stability throughout development and commercialization.

## Figures and Tables

**Table 1:** Chromane and amide dihedral angles (°) of the lowest-energy TPZ conformers in solutions and in Polymorphs A and B.

Angle Type	Energy Minimum in CHCl_3_		Energy Minimum in Water		Individual Conformers in Polymorphs A and B			
Dihedral angle (°)	TPZ-Tautomer				TPZ_Polymorph			
Torsion type	1	2	1	2	A1	A2	B1	B2
Chromane side	−72.92 (287.08)	−170.09 (189.91)	−73.56 (286.44)	91.94	168.71	55.05	−11.61 (348.39)	3.40
Amide side	−44.42 (315.58)	−142.43 (217.57)	−46.96 (313.04)	−45.94 (314.06)	−127.91 (232.09)	114.63	−138.60 (221.40)	134.03

Note: Dihedral angles are reported on a −180° to +180° scale, with equivalent 0–360° values shown in parentheses.

**Table 2:** Relative single-point energy differences (ΔE, kcal/mol) among hydrogen-bonded dimers in TPZ polymorphs A and B, calculated at the DFT-D [wB97X-D3(BJ)/def2-TZVPP] level.

Polymorph Type	TPZ_Polymorph A		TPZ_Polymorph B	
Hydrogen-bonding type	1	2	1	2
Δ*E* (kcal/mol)	2.49	3.37	0.00	12.44

Note: The lowest single-point energy among all calculated dimers was set as the zero-energy reference (TPZ_PolymorphB_1). The Δ*E* values represent relative energy differences for the hydrogen-bonded dimers shown in Figure 6.

**Table 3:** Solubility of Polymorph A, Polymorph B, and amorphous TPZ in acetone, methanol, and water at 20.0 ± 0.5 °C.

Solvent	Polymorph A (mg/mL)	Polymorph B (mg/mL)	Amorphous (mg/mL)
Acetone	10	520	660
Methanol	257	552	580
Water	0.035	0.116	0.600

Note: The values represent the measured concentrations after 1 min of stirring under supersaturated conditions, determined using HPLC.

**Table 4:** Kinetic parameters (rate constant *k* and Avrami exponent *n*) from KJMA fits of TPZ phase transformations in different solvents.

Solvent	KJMA Equation	Rate Constant (*k*)	Avrami Exponent (*n*)
Acetone	*Y* = 1 − exp(−0.932*t*^5.5^)	0.932	5.5
Methanol	*Y* = 1 − exp(−12.76*t*^1.24^)	12.76	1.24
Water	*Y* = 1 − exp(−4.28 × 10^−9^*t*^6.3^)	4.28 × 10^−9^	6.3

Note: The rate constant (*k*) is reported in *h*^−^*ⁿ*, and the Avrami exponent (*n*) reflects the dimensionality and mechanism of the transformation. Parameters were obtained by the nonlinear regression of HR-PXRD-derived transformation profiles using the KJMA equation.

**Table 5:** Kinetic parameters of the SMPT from amorphous to Polymorph A in three solvents, estimated using the KJMA equation.

Solvent	KJMA Equation	Rate Constant (*k*)	Avrami Exponent (*n*)
Acetone	*Y* = 1 − exp(−1.84 × 10^−3^*t*^8.42^)	1.84 × 10^−3^	8.42
Methanol	*Y* = 1 − exp(−0.22*t*^11.3^)	0.22	11.3
Water	*Y* = 1 − exp(−3.94 × 10^−28^*t*^11.5^)	3.94 × 10^−28^	11.5

Note: All *k* values were derived using time in hours. DSC was used for water.

## Data Availability

Data is contained within the article or supplementary material.

## Supplementary Materials

The following supporting information can be downloaded at: https://www.mdpi.com/article/10.3390/pharmaceutics17070928/s1, Section S1. Existing data. Figure S1. The phase transitions of Tegoprazan Polymorph B under accelerated stability testing conditions were monitored over time using X-ray diffraction (XRD). Figure S2. The phase transitions of amorphous Tegoprazan under accelerated stability testing conditions were monitored over time using X-ray diffraction (XRD). Figure S3. DSC measurements were performed on Tegoprazan Polymorph B at weeks 1, 3, 5, and 8. Figure S4. DSC measurements were performed on amorphous Tegoprazan at weeks 1, 3, 5, and 8. Section S2. KJMA analysis and calibration curve with 95% CI and PI for amorphous-to-Polymorph A SMPT. Figure S5. Integrated intensity comparison between low-angle (5–35°) and high-angle (35–70°) regions for pure Polymorph A and B. Figure S6. Powder X-ray diffraction (PXRD) patterns of pure Polymorph A and B reference samples for SMPT quantification. Figure S7. Powder X-ray diffraction (PXRD) pattern of pure Polymorph A, highlighting the specific diffraction peaks (Range 0, Range 1, and Range 2) used for quantitative analysis. Document S1. Description of confidence and prediction interval calculation. Document S2. Determination of LOD and LOQ. Table S1. Calibration performance metrics for PXRD-based quantification of Tegoprazan crystallinity. Document S3. Calibration analysis report (with confidence and prediction intervals) for acetone. Figure S8. Calibration curve for the new dataset with quadratic fit, 95% confidence interval, and 95% prediction interval. Document S4. KJMA analysis report. Document S5. Calibration analysis report (with confidence and prediction intervals) for methanol. Figure S9. Calibration curve for the batch dataset with quadratic fit, 95% confidence interval, and 95% prediction interval. Document S6. KJMA analysis report: SMPT experiment in methanol. Document S7. Interpretation of KJMA analysis for SMPT experiment in water. Table S2. Time-dependent ΔH values and normalized conversion for the phase transformation of Tegoprazan Polymorph A in water (reference ΔH = 75.594 J/g). Section S3. Solubility and SMPT analysis by KJMA equation. (Extended) Table S3. Solubility of Tegoprazan Polymorphs A and B and the amorphous form in acetone, methanol, and water and Gibbs free energy of solution for Polymorphs A and B. Table S4. KJMA analysis of the phase transformation from Tegoprazan Polymorph B to Polymorph A in various solvents. Figure S10. Comparison of SMPT in various solvents for Tegoprazan: transformation from Polymorph B to Polymorph A. Figure S11. SMPT of Tegoprazan in MEK: time-dependent PXRD analysis and kinetic profiles. Figure S12. Time-dependent PXRD patterns of Tegoprazan during phase transformation in various solvents. Figure S13. HPLC chromatograms of TPZ polymorphs and amorphous form.

## Abbreviations

The following abbreviations are used in this manuscript:

API	Active Pharmaceutical Ingredient;
CI	Confidence Interval;
CSP	Crystal Structure Prediction;
DFT-D	Density Functional Theory with Dispersion Correction;
DSC	Differential Scanning Calorimetry;
HPLC	High-Performance Liquid Chromatography;
HR-PXRD	High-Resolution PXRD;
ICH Q2(R1)	International Council for Harmonisation Guideline Q2(R1): Validation of Analytical Procedures;
KJMA	Kolmogorov–Johnson–Mehl–Avrami;
LOD	Limit of Detection;
LOQ	Limit of Quantitation;
NMR	Nuclear Magnetic Resonance;
NOE	Nuclear Overhauser Effect;
OLS	Ordinary Least Squares;
OPLS4	Optimized Potentials for Liquid Simulations 4;
PCM	Polarizable Continuum Model;
PI	Prediction Interval;
P-CAB	Potassium-Competitive Acid Blocker;
PXRD	Powder X-ray Diffraction;
ROESY	Rotating-Frame Overhauser effect spectroscopy;
R_wp_	Weighted Profile R-factor;
R_exp_	Expected R-factor;
SDPD	Structure Determination by Powder Diffractometry;
SMPT	Solvent-Mediated Phase Transformation;
TGA	Thermogravimetric Analysis;
TPZ	Tegoprazan;
Z′	Number of independent molecules in asymmetric unit (crystallography term).
