# Supplementary material for: Comprehensive Investigation of Polymorphic Stability and Phase Transformation Kinetics in Tegoprazan
Source: Pharmaceutics. 2025 Jul 18;17(7):928. doi: 10.3390/pharmaceutics17070928 (PMC12300558; doi:10.3390/pharmaceutics17070928)
Supplement: Supplementary file 1 [file pharmaceutics-17-00928-s001.zip › pharmaceutics-3736914-supplementary.pdf]

Section S1. Existing data

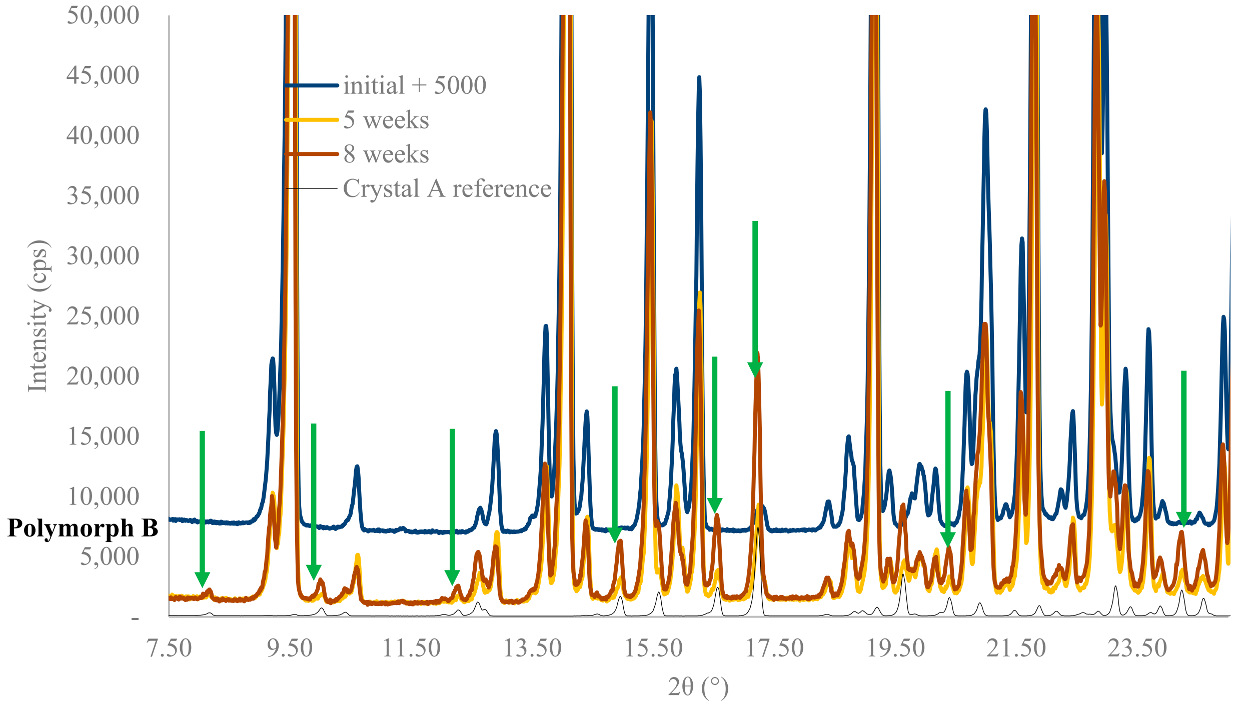

Figure S1. The phase transitions of Tegoprazan Polymorph B under accelerated stability testing conditions were monitored over time using X-ray diffraction (XRD). The intensity of the arrows increases over time. Intensities are shown in cps (counts per second), representing detector response. A vertical offset was applied to the "initial" profile (+5000 cps) for clarity. This figure was prepared with reference to the supplementary materials of [20].

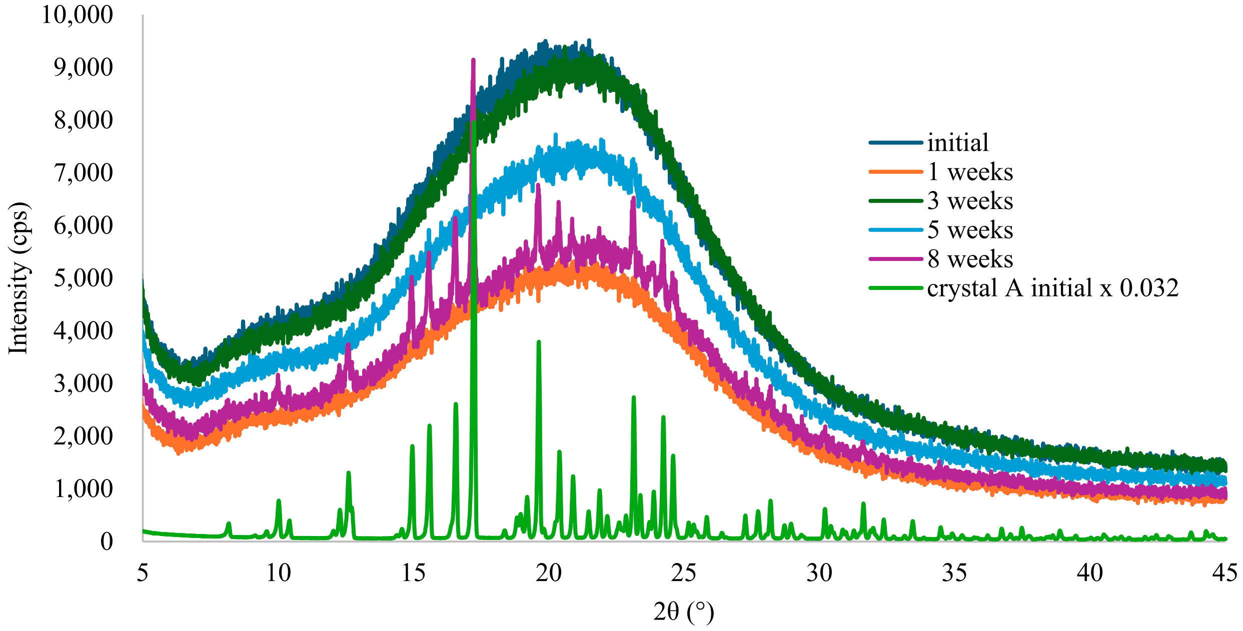

Figure S2. The phase transitions of amorphous Tegoprazan under accelerated stability testing conditions were monitored over time using X-ray diffraction (XRD). "Crystal A initial" was scaled by a factor of 0.032 to match the intensity range of amorphous-phase samples for easier comparison.

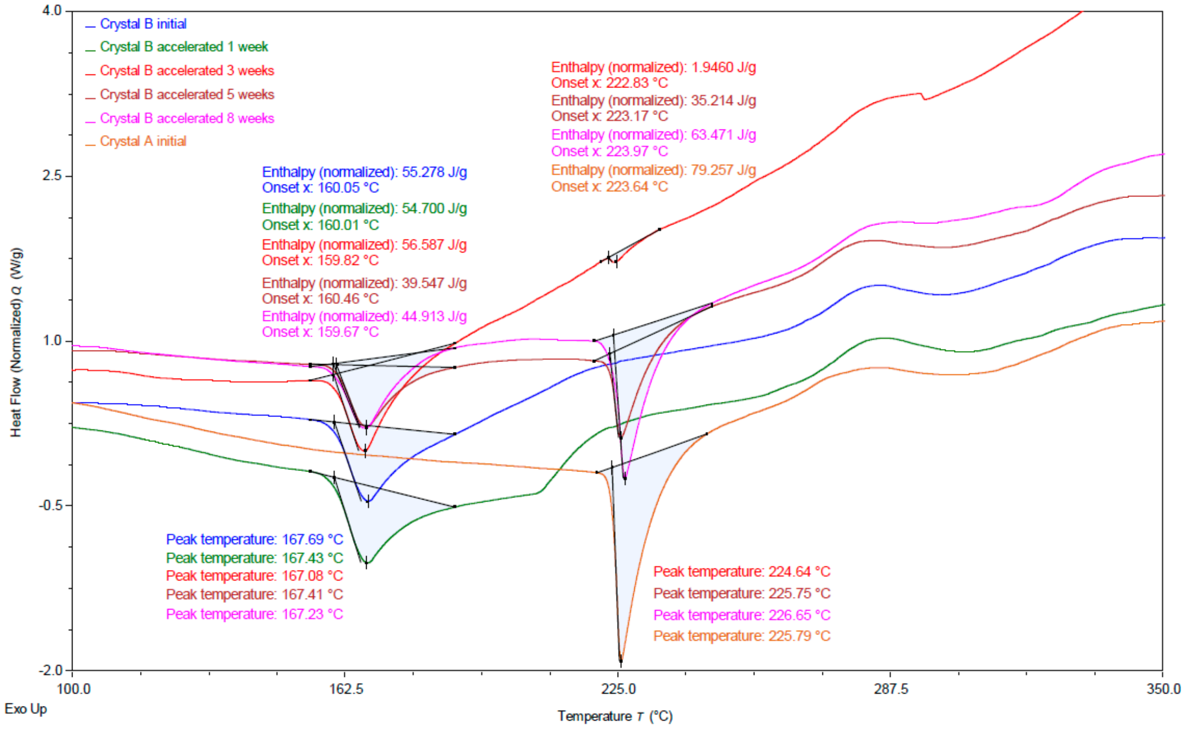

Figure S3. DSC measurements were performed on Tegoprazan Polymorph B at weeks 1, 3, 5, and 8. A new endothermic peak was observed at the same temperature as Polymorph A, appearing over time. The intensity of the arrows increases over time. This figure was prepared with reference to the supplementary materials of [20].

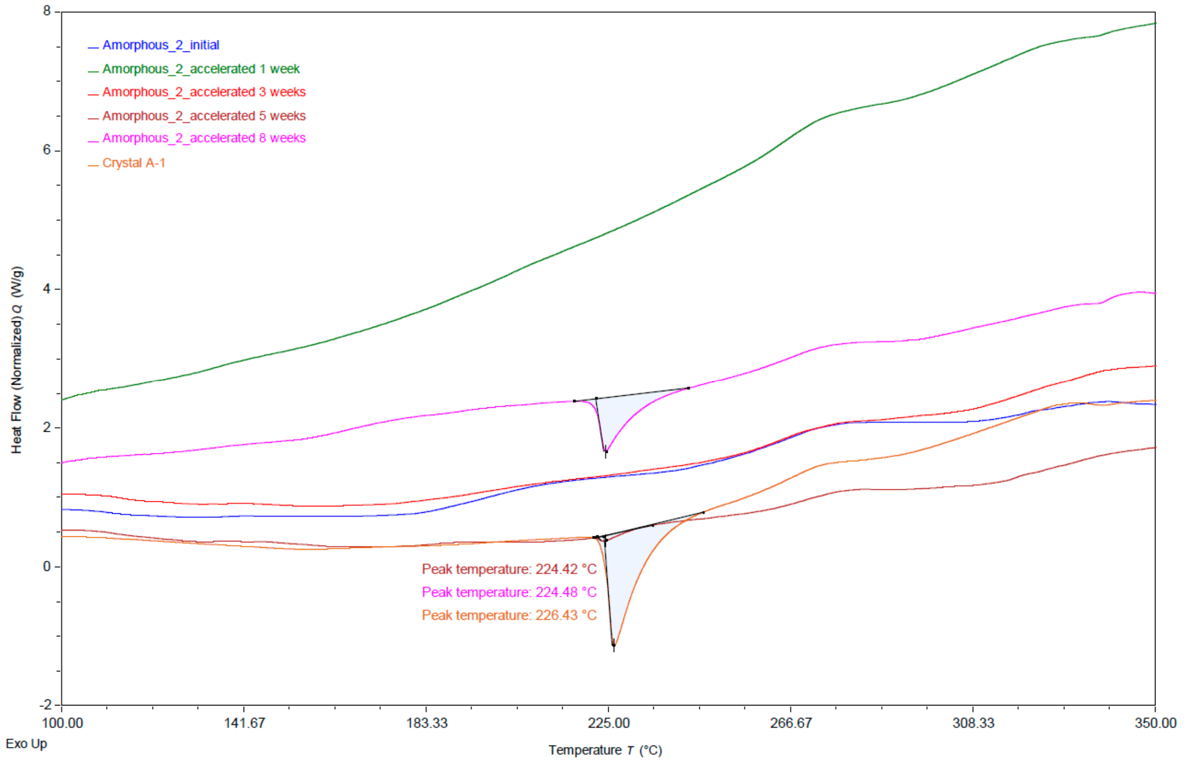

Figure S4. DSC measurements were performed on Amorphous Tegoprazan at weeks 1, 3, 5, and 8. A new endothermic peak was observed at the same temperature as Polymorph A, appearing overtime.

Section S2. KJMA Analysis and Calibration Curve with 95% CI and PI for Amorphous-to-Polymorph A SMPT

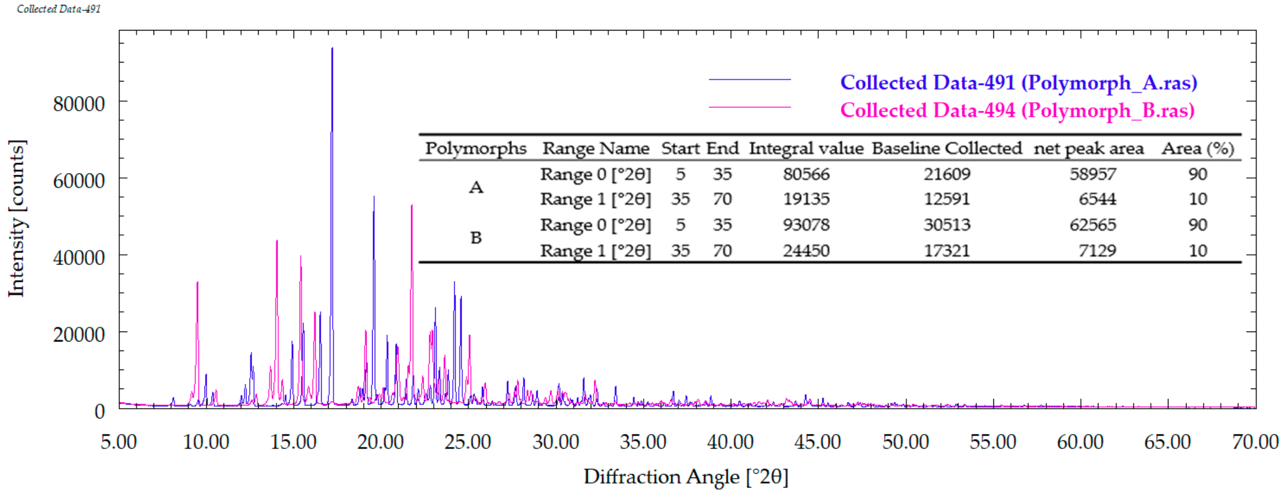

Figure S5. Integrated intensity comparison between low-angle (5°–35°) and high-angle (35°–70°) regions for pure Polymorph A and B. PXRD patterns of Polymorph A and B measured over 5°–70° with integrated intensity values summarized in the table. The results confirm that ~90% of total intensity is contained within the 5°–35° range, justifying its use for time-sensitive SMPT monitoring.

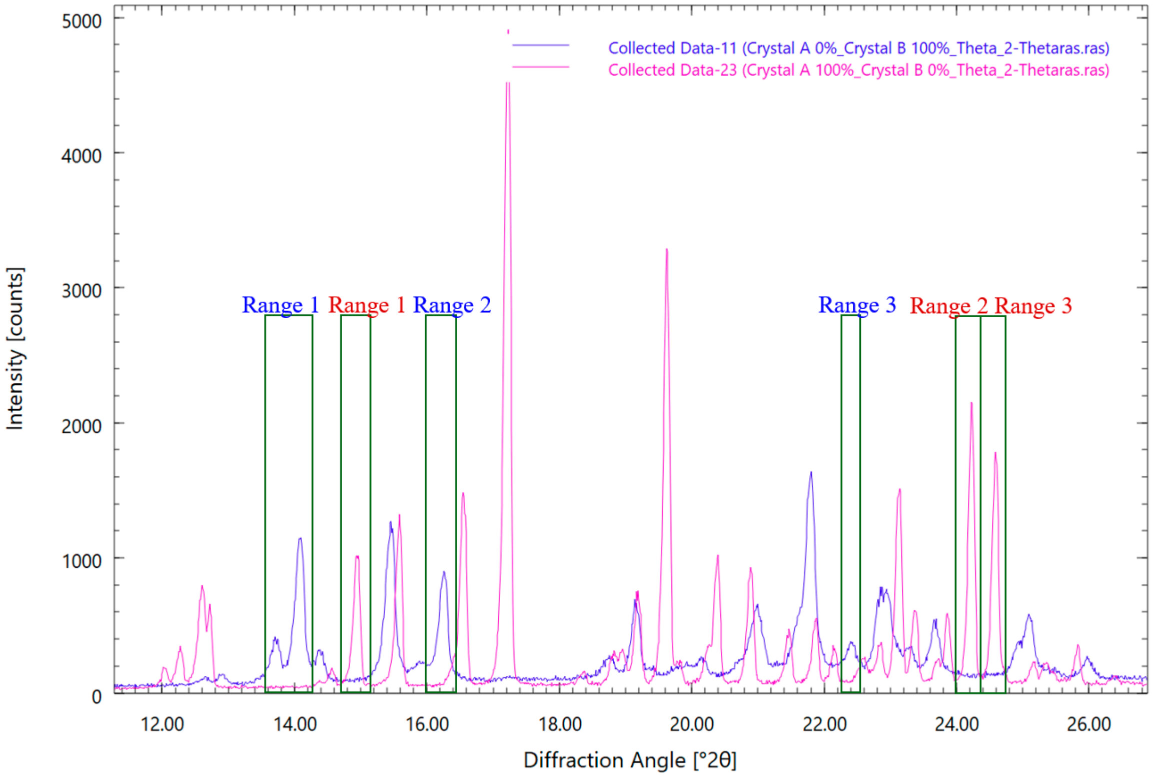

Figure S6. Powder X-ray diffraction (PXRD) patterns of pure Polymorph A and B reference samples for SMPT quantification. Overlay of PXRD patterns for pure Polymorph A (100%) and pure Polymorph B (100%) used as reference profiles in the solid-state transformation (SMPT) analysis. Non-overlapping characteristic peaks for each polymorph were selected (Range 1–3) to calculate the fractional concentrations of Polymorph A and B during transformation experiments.

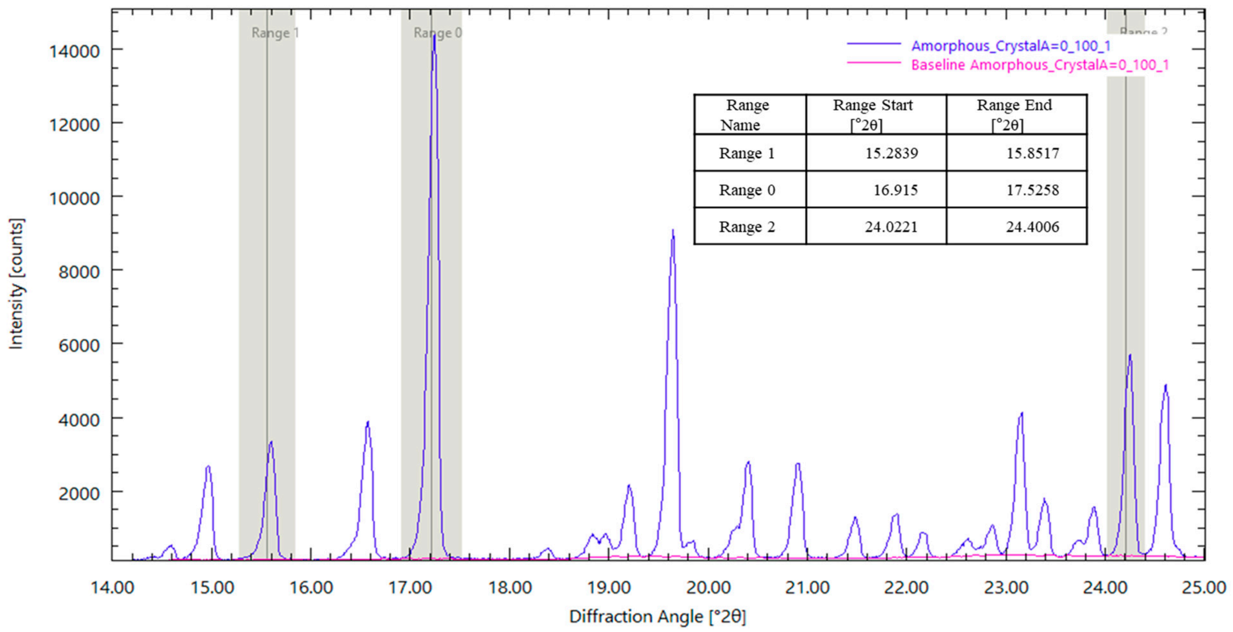

Figure S7. **Powder X-ray diffraction (PXRD) pattern of pure Polymorph A**, highlighting the specific diffraction peaks (Range 0, Range 1, and Range 2) used for quantitative analysis. The shaded regions correspond to the defined peak ranges, and the accompanying table lists the exact 2θ start and end positions for each range. The blue line shows the measured diffraction pattern of 100% Polymorph A, while the magenta line indicates the baseline. To perform quantitative phase analysis, specific diffraction peak regions were selected from the PXRD pattern of pure Polymorph A. These regions (Range 0, Range 1, and Range 2) were identified based on their distinct peak shapes and intensities and were highlighted for integration. The start and end positions (in 2θ) were precisely defined to ensure consistent data extraction. The measured PXRD pattern (blue line) and the baseline (magenta line) are shown, providing a clear visualization of the reference peak ranges used for calibration and subsequent quantification.

Document S1. Description of Confidence and Prediction Interval Calculation

To evaluate the uncertainty in the calibration model, we calculated both 95% confidence intervals (CIs) and 95% prediction intervals (PIs) using an ordinary least squares (OLS) regression approach. All calculations were carried out in Python (v3.11) using the statsmodels package (v0.14.0). The calibration curve was modeled as a second-order polynomial (quadratic) regression, which includes both the linear and squared terms of crystallinity (%) as predictors. The model equation is given by the following:

$$Y=\beta_0+\beta_1X+\beta_2X^2+\varepsilon$$

Here, Y represents the measured intensity; X is the crystallinity (%); β_0, β_1, and β_2 are the regression coefficients estimated from the data; ε is the random error (residual).The 95% confidence interval (CI) indicates the range in which we expect the average intensity value (mean response) to lie for a given level of crystallinity. It reflects the uncertainty associated with estimating the regression curve itself. The 95% prediction interval (PI), on the other hand, is wider than the CI. It shows the range within which we expect a single new measurement to fall, given a specific crystallinity value. This accounts not only for uncertainty in the regression model but also for natural variability in individual observations.

Document S2. Determination of LOD and LOQ

To determine the detection and quantification limits for the PXRD-based analysis, 15 replicate measurements of 100% amorphous tegoprazan samples (corresponding to 0% Crystal A content) were performed. The standard deviation of the blank measurements was calculated and combined with the sensitivity of the calibration curve at the low-concentration end (determined as the derivative of the quadratic regression equation at x = 0) to compute the limits. The limit of detection (LOD) was defined as

$$LOD = 3 \times \frac{standard\ deviation\ (blank)}{sensitivity}$$

and the limit of quantification (LOQ) was defined as follows:

$$LOQ = 10 \times \frac{standard\ deviation\ (blank)}{sensitivity}$$

These limits were incorporated into the quantitative analysis template to ensure accurate phase quantification within validated ranges.

Table S1. Calibration performance metrics for PXRD-based quantification of Tegoprazan crystallinity.

Metric	Value
Standard deviation (blank)	5.0
Sensitivity (low end)	15.7
LOD (%)	0.96
LOQ (%)	3.21

Document S3. Calibration Analysis Report (with Confidence and Prediction Intervals) for Acetone

1. Calibration Data Summary

This section summarizes the calibration data for the provided new dataset. The data includes actual Polymorph A (%) values, measured total intensities, normalized percentages (scaled to a maximum of 2704.7 → 100%), and predicted percentages from the quadratic model fitting. This study was conducted in acetone as the solvent.

2. Model Coefficients

The fitted quadratic equation is
Y = 3.8314e-03 * X◎+ 5.7917e-01 * X + 7.5507e-01
with an R◎value of 0.9971.

These coefficients were determined using a second-degree polynomial fit. The high R◎ indicates a strong agreement between the fitted model and the normalized calibration data.

3. Calibration Plot with Intervals

The figure below shows the calibration plot with predicted values, quadratic fit, and the 95% confidence and prediction intervals.

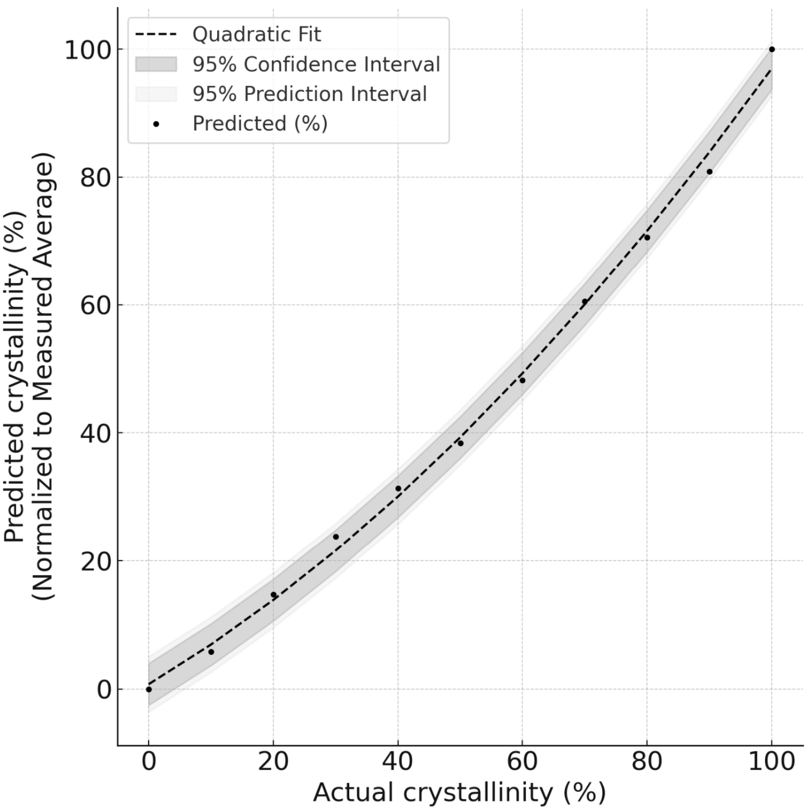

Figure S8. Calibration curve for the new dataset with quadratic fit, 95% confidence interva l, and 95% prediction interval.

4. Auto Calculation Template

An Excel template named "New_Calibration_Template_FIXED.xlsx" is provided. It contains three sheets:

- Calibration Data: Raw and normalized data with model predictions.
- Model Coefficients: Extracted quadratic coefficients and R◎value.
- SMPT Experiment Calculation: A ready-to-use template where users can input Polymorph A (%) values and obtain predicted normalized values automatically.

Ensure new measurement inputs are scaled using the same normalization reference (2704.7 → 100%) for consistent predictions.

5. Usage Notes

This calibration framework is designed for quantitative phase analysis in SMPT experiments. When applying to new data, users should verify the measurement conditions, instrument settings, and normalization standards to ensure the validity of predicted outputs. For advanced confidence and prediction interval calculations, refer to the graphical outputs generated alongside this report. This study was conducted in acetone as the solvent.

Document S4. KJMA Analysis Report: SMPT Experiment in Acetone

This report presents the Kolmogorov–Johnson–Mehl–Avrami (KJMA) kinetic analysis of the time-dependent formation of Polymorph A (%) in a solvent-mediated phase transformation (SMPT) experiment conducted using acetone as the solvent. The analysis focuses on the 1.5–2 h interval, where the most pronounced transformation kinetics were observed. Derived parameters were

applied to the entire time course to assess overall agreement. The kinetic parameters (n and k) were initially estimated by applying the KJMA equation to selected portions of the experimental data based on the theoretical model assumptions. Subsequently, n and k values were manually adjusted to optimize the fit across the entire experimental dataset, ensuring consistency between the model and the observed transformation behavior.

1. Fitted Parameters (1.5–2 h Subset, Acetone)

Avrami exponent (n): 8.42

Rate constant (k): $1.84e^{-3}$

2. Interpretation

The Avrami exponent (n ≈ 8.42) suggests a complex nucleation and growth process in the acetone system, possibly involving multi-dimensional growth or time-dependent nucleation mechanisms. The small value of the rate constant (k ≈ $1.84e^{-3}$) indicates that the transformation proceeds slowly under the tested conditions. The focused fitting in the 1.5–2 h window allows the precise estimation of kinetic parameters, which were then used to generate a theoretical fit over the full time course. This approach provides insight into the dynamic progression of the phase transformation in acetone and the effectiveness of the KJMA model for describing the observed behavior.

Document S5. Calibration Analysis Report (with Confidence and Prediction Intervals) for Methanol

1. Calibration Data Summary

This section summarizes the calibration data for the provided batch dataset. The data include actual Polymorph A (%) values, measured total intensities, normalized percentages (scaled to a maximum of 2341.1 → 100%), and predicted percentages from the quadratic model fitting. This study was conducted in methanol as the solvent.

2. Model Coefficients

The fitted quadratic equation is
Y = 2.8447e-03 * X◎+ 7.2046e-01 * X + 5.8110e-01
with an R◎value of 0.9954.

These coefficients were determined using a second-degree polynomial fit. The high R◎ indicates a strong agreement between the fitted model and the normalized calibration data.

3. Calibration Plot with Intervals

The figure below shows the calibration plot with predicted values, quadratic fit, and the 95% confidence and prediction intervals.

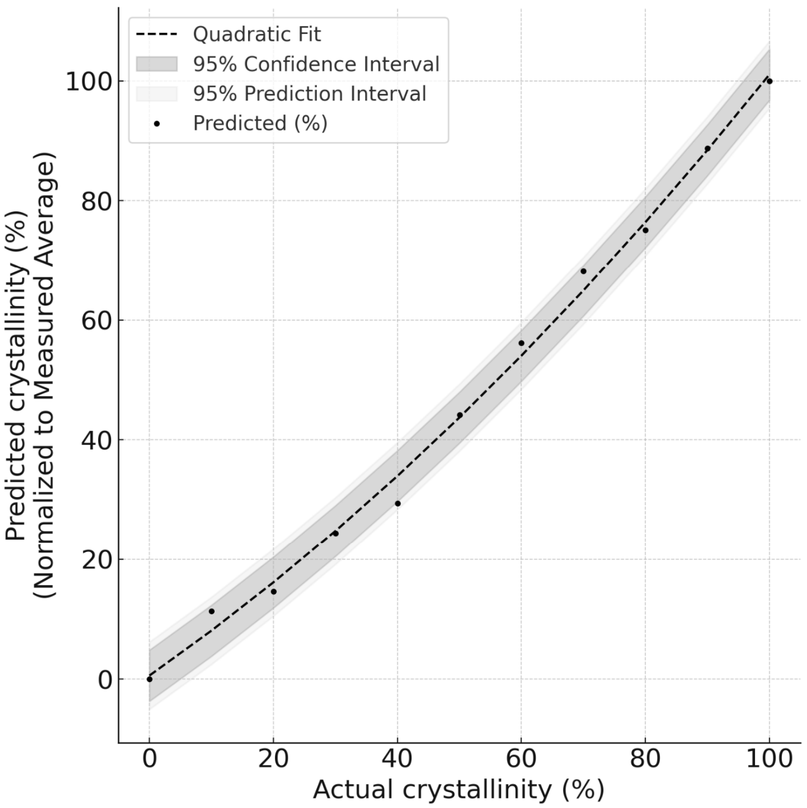

Figure S9. **Calibration curve for the batch dataset with quadratic fit, 95% confidence interval, and 95% prediction interval.**

4. Auto Calculation Template

An Excel template named "Batch_Calibration_Template.xlsx" is provided. It contains three sheets:

- Calibration Data: Raw and normalized data with model predictions.
- Model Coefficients: Extracted quadratic coefficients and R⊚value.
- SMPT Experiment Calculation: A ready-to-use template where users can input Polymorph A (%) values and obtain predicted normalized values automatically.

Ensure that new measurement inputs are scaled using the same normalization reference (2341.1 → 100%) for consistent predictions.

5. Usage Notes

This calibration framework is designed for quantitative phase analysis in SMPT experiments. When applying to new data, users should verify the measurement conditions, instrument settings, and normalization standards to ensure the validity of predicted outputs. For advanced confidence and prediction interval calculations, refer to the graphical outputs generated alongside this report. This study was conducted in methanol as the solvent.

Document S6. KJMA Analysis Report: SMPT Experiment in Methanol

This report summarizes the KJMA (Kolmogorov–Johnson–Mehl–Avrami) analysis performed on the crystallization data of Polymorph A in methanol. The analysis focused on estimating the Avrami exponent (n) and the rate constant (k) over the localized time window between 35 and 55 minutes and applying these parameters to fit the full dataset. The kinetic parameters (n and k) were initially estimated by applying the KJMA equation to selected portions of the experimental data based on the

theoretical model assumptions. Subsequently, *n* and *k* values were manually adjusted to optimize the fit across the entire experimental dataset, ensuring consistency between the model and the observed transformation behavior.

1. Fitted Parameters (1.5–2 h Subset, Methanol)

Avrami exponent (*n*): 11.3

Rate constant (*k*): 0.22

2. Interpretation

The Avrami exponent (*n*) was determined to be 11.3, indicating the nature of the crystallization mechanism and growth dimensionality. A higher value typically suggests more complex nucleation and growth behaviors. The rate constant (*k*) was found to be 0.22, representing the overall speed of the phase transformation. These parameters, derived from the localized time window, can provide mechanistic insights into the solvent-mediated phase transformation (SMPT) process and help predict the time evolution of the system under methanol conditions.

Document S7. Interpretation of KJMA Analysis for SMPT Experiment in Water

1. Overview

This analysis applies the Kolmogorov–Johnson–Mehl–Avrami (KJMA) equation to data obtained from a solvent-mediated phase transformation (SMPT) experiment in water. The goal is to quantify the phase transition kinetics and understand the underlying mechanism, using differential scanning calorimetry (DSC)-derived data.

2. Experimental Details

Differential scanning calorimetry (DSC) measurements were conducted using a TA Instruments SD T650 system (TA Instruments, New Castle, DE, USA). Approximately 3–8 mg of each sample was placed in an open alumina pan (90 μL). The samples were equilibrated at 30.00°C and then heated from 30°C to 300°C at a rate of 10°C/min under a nitrogen atmosphere with a flow rate of 20 mL/min. The obtained data were processed using Trios software (version 5.7.0.56, TA Instruments). For normalization, the ΔH value of fully crystallized Polymorph A measured under identical conditions (with or without immersion in water) was used as the 100% reference.

3. Procedure and Results

- The KJMA equation was linearized:

$$\ln[-\ln(1 - Y(t))] = \ln k + n \ln t$$

- From selected experimental data points (8th, 9th, 10th, and 11th day), the slope (*n*) and intercept (ln *k*) were determined.
- Estimated parameters:

  $n \approx 11.5$

  $k \approx 3.94 \times 10^{-28}$

- Applying these parameters across the entire time range (3–17 days) showed excellent agreement in the central growth region, with expected deviations during the initial lag phase and final saturation. The kinetic parameters (*n* and *k*) were initially estimated by applying the KJMA equation to selected portions of the experimental data, based on the theoretical model assumptions. Subsequently, *n* and *k* values were manually adjusted to optimize the fit across the entire experimental dataset, ensuring consistency between the model and the observed transformation behavior.

4. Interpretation

- The obtained n value indicates the dimensionality and complexity of the transformation process:

$n \approx 1 \rightarrow$ linear growth.

$n \approx 2–3 \rightarrow$ random nucleation and spherical growth.

$n \gg 3 \rightarrow$ complex multi-step processes or hindrances.

- Our result ($n \approx 11.5$) suggests a highly complex transformation that is likely influenced by solvent effects, nucleation barriers, or multi-phase interactions.
- The very low k value supports the observation that the transformation progresses over days, not hours, highlighting the slow kinetics in aqueous systems.

5. Implications

- The SMPT to Polymorph A is not a simple one-step process but shows complex kinetics requiring targeted kinetic analysis.
- The KJMA model fits well when applied selectively to the main transformation window; including plateau regions (initial or final) reduces accuracy.
- These insights underscore the importance of careful data selection and normalization when modeling solid-state phase transformations using DSC-derived data.

Table S2. Time-dependent ΔH values and normalized conversion of the phase transformation of Tegoprazan Polymorph A in water (reference ΔH = 75.594 J/g).

Time (days)	ΔH (J/g)	normalized ΔH (%)	Time (days)	ΔH (J/g)	normalized ΔH (%)
0.000	0.000	0.0	11.069	70.710	93.9
3.000	0.000	0.0	11.271	73.275	97.3
5.271	0.405	0.5	11.326	70.322	93.4
6.406	1.210	1.6	12.069	74.353	98.7
7.326	0.883	1.2	12.406	72.504	96.3
8.069	6.422	8.5	13.326	72.169	95.8
9.406	53.901	71.6	14.069	74.977	99.6
10.326	62.134	82.5	15.069	75.311	100.0
10.406	65.913	87.5	17.750	74.284	98.6

6. Future Work

- Apply similar analyses to other solvent systems (e.g., acetone, methanol) for comparative kinetic profiling.
- Explore alternative kinetic models (e.g., Avrami–exponential, two-phase models) for cross-validation.
- Conduct sensitivity analyses on experimental parameters (temperature, concentration, and particle size) to better understand kinetic drivers.

Section S3. Solubility and SMPT Analysis by KJMA Equation (Extended)

Table S3. Solubility of Tegoprazan Polymorphs A and B and the amorphous form in acetone, methanol, and water and Gibbs free energy of the solution for Polymorphs A and B.

Solvent	Polymorph A		Polymorph B		Amorphous	
	Solubility	$\Delta G_{solution}$	Solubility	$\Delta G_{solution}$	Solubility	$\Delta G_{solution}$
	mg/mL	kJ/mol	mg/mL	kJ/mol	mg/mL	kJ/mol
Acetone	10	-4.99	520	-14.77	660	–
Methanol	257	-13.04	552	-14.92	580	–
Water	0.035	9.02	0.116	6.07	0.600	–

Table S4. KJMA analysis of the phase transformation from Tegoprazan Polymorph B to Polymorph A in various solvents.

Solvents	The KJMA equations
Methanol	$Y_{Methanol} = 1 - e^{-12.78t^{1.24}}$
2-Propanol	$Y_{Acetone} = 1 - e^{-0.932t^{5.5}}$
Acetone	$Y_{2-Propanol} = 1 - e^{-1.111\times10^{-2}t^{4.8}}$
Toluene	$Y_{Toluene} = 1 - e^{-4.26\times10^{-19}t^{16.5}}$
Water	$Y_{Water} = 1 - e^{-4.28\times10^{-8}t^{6.3}}$

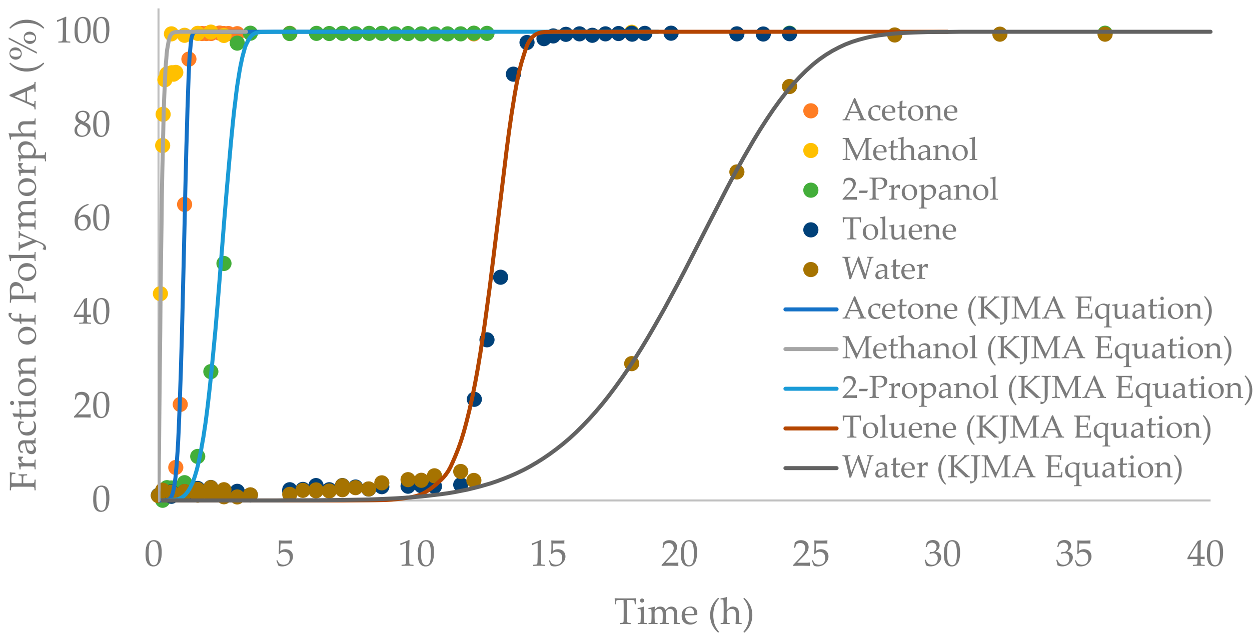

Figure S10. Comparison of SMPT in various solvents for Tegoprazan: transformation from Polymorph B to Polymorph A.

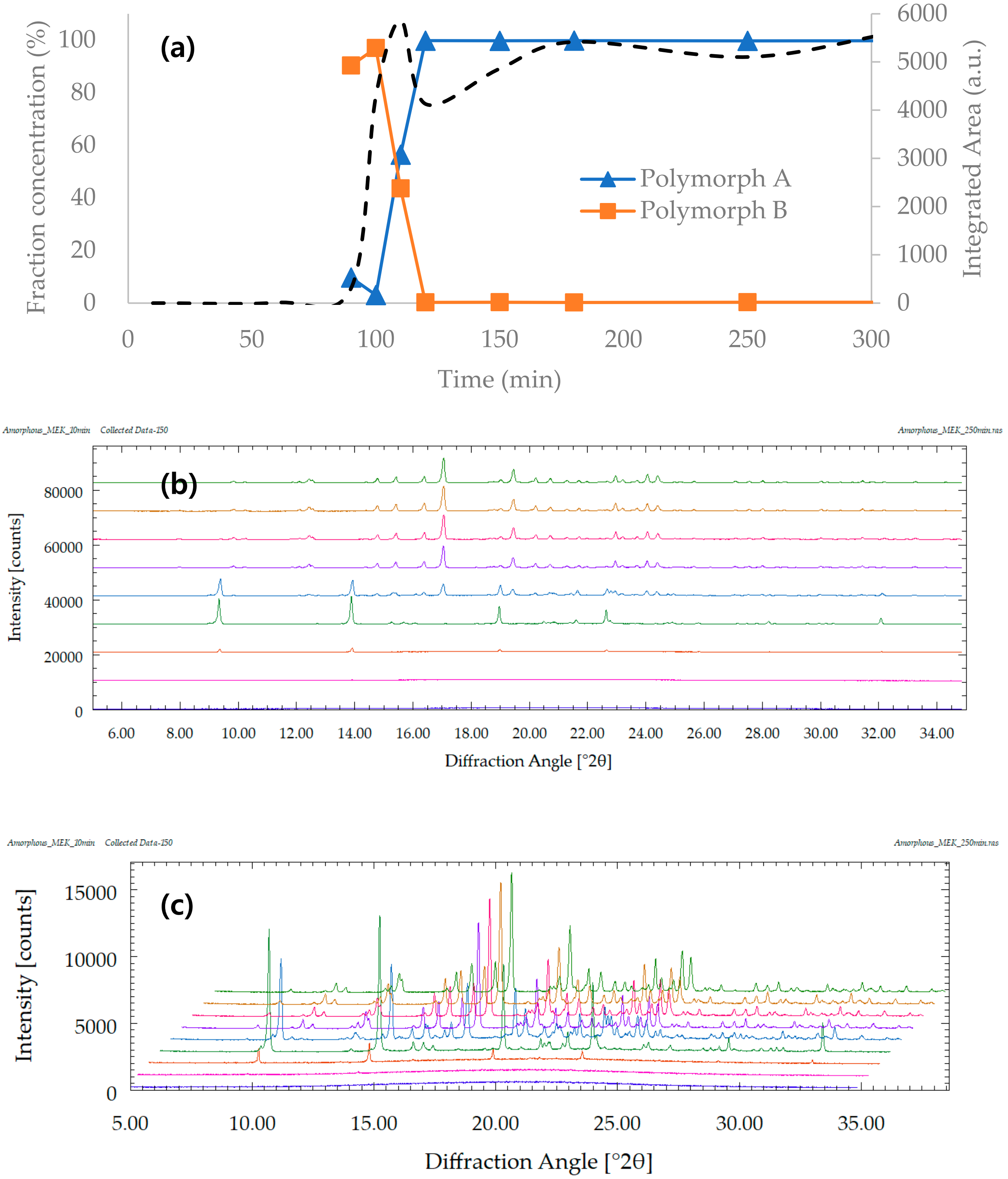

Figure S11. Solvent-mediated phase transformation of Tegoprazan in MEK: time-dependent PXRD analysis and kinetic profiles. (a) Time-dependent phase fraction profiles of Polymorph A (▲) and Polymorph B (■) during the phase transformation in 2-butanone ketone (MEK), as determined by Rietveld refinement. The dashed line represents the integrated area of the characteristic diffraction peak. (b) Stacked PXRD patterns collected at multiple time points during the transformation process. The intensities are offset for clarity. (c) Three-dimensional representation of the PXRD patterns shown in (b).

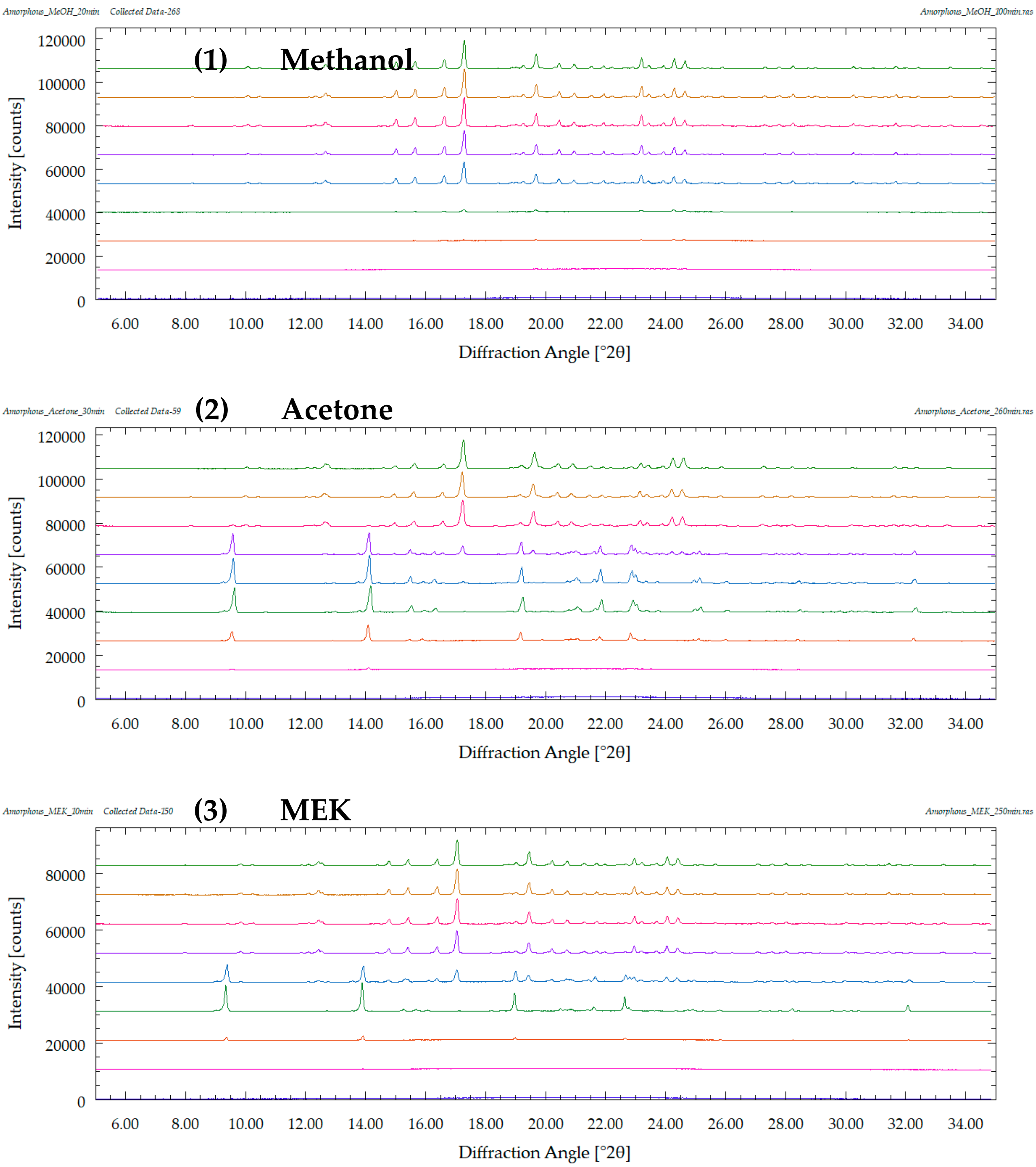

Figure S12. Time-resolved PXRD patterns of Tegoprazan during phase transformation in various solvents. Time-dependent PXRD patterns collected during the phase transformation experiments of Tegoprazan in (1) methanol, (2) acetone, and (3) MEK. Patterns were acquired at multiple time points and vertically offset for clarity. The progressive changes in diffraction peak intensities reflect the dynamic conversion from the initial phase to the thermodynamically stable polymorph.

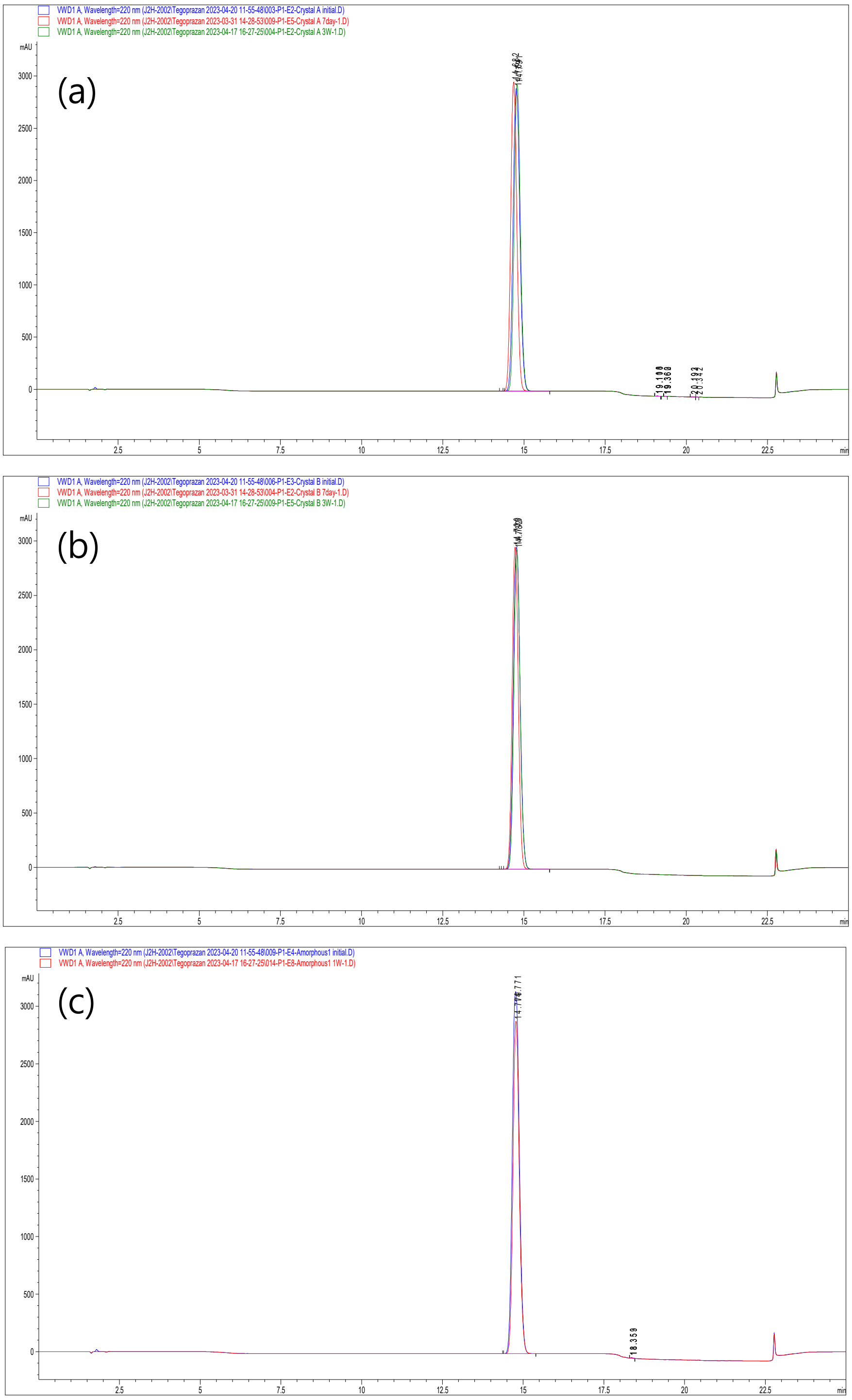

Figure S13. HPLC chromatograms of TPZ polymorphs and amorphous form. (a) Polymorph A, (b) Polymorph B, and (c) amorphous TPZ. All samples were analyzed under identical HPLC conditions using detection at 220 nm. The chromatograms exhibit nearly identical retention times (~14.7 min), indicating that no significant differences in chromatographic behavior exist among the three solid-state forms. These data confirm that HPLC cannot distinguish between the polymorphic and amorphous forms of TPZ under the applied analytical conditions, though it remains suitable for the quantification of the API.

The Supplementary Materials provide additional experimental details, figures, and data that support the results and discussions presented in the main manuscript.
